# Modular Assembly of Dynamic Polymer Networks From Heteroaffinity Cross‐Links to Multivalent Proteins

**DOI:** 10.1002/anie.202526058

**Published:** 2026-02-05

**Authors:** Tianyue Dai, Yuntao Qiu, Katherine M. Leon Hernandez, Wesley Chan, Katrina Mejia, Natalia Nemeria, Colin D. Kinz‐Thompson

**Affiliations:** ^1^ Department of Chemistry Rutgers University‐Newark Newark New Jersey USA; ^2^ The Advanced Science Research Center Graduate Center of the City University of New York New York New York USA

**Keywords:** biophysics, NMR spectroscopy, nonequilibrium processes, polymers, self‐assembly

## Abstract

Dynamic polymer networks (DPN) leverage transient cross‐linking to yield macroscopic materials that exhibit self‐healing and environmentally responsive behaviors. Despite the broad scope of chemical interactions available for cross‐linking, imbuing those transient interactions into biologically compatible materials is difficult because many chemistries are incompatible with aqueous conditions, and it is difficult to tune preexisting biological interactions that have evolved over millions of years for specificity. To enable the assembly of chemically tunable and biologically compatible DPNs, we have developed a set of bifunctional, heteroaffinity cross‐linkers (HAX) where the reactive moieties have different binding affinities to the same binding sites of an oligomeric protein. The use of cross‐linking moieties with vastly different dissociation rates enables purification of protein modules with monodisperse HAX valencies. Assembly of DPNs from stoichiometrically identical pairs of protein modules then yields unique, metastable, nonequilibrium network topologies. Here, we demonstrate these concepts using the well‐studied avidin‐biotin interaction chemistry. We also develop a pH‐sensitive HAX that yields DPNs with robust pH‐responsive assembly dynamics, and demonstrate how this DPN can be made into a magnetically responsive, molecular delivery system to low‐pH regions, such as tumor microenvironments.

## Graphical abstract

This study develops a method to modularly assemble oligomeric binding proteins, like streptavidin, using heterobifunctional cross‐linkers that have both a strong‐ and a weak‐affinity binding moiety. These dynamic cross‐linkers enable the assembly of polymer networks with unique meta‐stable nonequilibrium topologies, and that exhibit chemically tunable and stimulus‐responsive dynamics.

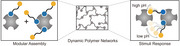

## 1 Introduction

The broad diversity of covalent cross‐linking reactions has led to a long history of researchers developing static polymer networks into strong, thermostable, high‐utility materials—from vulcanized rubber to bakelite to epoxy resins [1, 2, 3]. Over the past few decades, the potential of noncovalent cross‐linking reactions to yield supramolecular polymer networks has also been realized in the quest to access materials with unique macroscopic properties [4, 5, 6, 7]. In these cases, the reactive cross‐linker moieties exist in a dynamic, dissociative equilibrium between a bound and an unbound state, both of which are accessible under ambient conditions due to a relatively low interaction energy—especially for noncovalent interactions. The relatively short lifetime of such a cross‐link yields a microscopically heterogeneous, dynamic polymer network (DPN) that has a well‐defined equilibrium structure and, consequently, has well‐defined macroscopic properties [8]. Notably, these properties imbue DPN‐based materials with self‐healing capabilities [9], and their macroscopic rheological properties can be drastically tuned by altering the affinity, valency, and/or density of the noncovalent cross‐linking reaction [3, 4, 5, 6, 7]. These properties have enabled the development of DPNs that respond to environmental stimuli [10] or that can act as a controlled drug delivery mechanism [11, 12].

While the diversity of DPN cross‐linking reactions spans organic, main group, and inorganic chemistries [13], several reactions with water‐compatible chemistries have found success integrating with biological systems [14]. For instance, biomolecules such as peptides have been used to create biocompatible hydrogels with tunable supramolecular and rheological properties [15, 16]. Notably, peptide‐based scaffolds have been shown to enable modular self‐assembly and tunability through covalent decoration prior to network formation [17, 18]. These biomolecular approaches, however, are generally top‐down designed using preexisting biological interactions, and consequently, fine‐tuning and generalizing such systems is difficult and often limited by functional biomolecular constraints [14]. As a result, while biocompatible DPNs show promise, they frequently lack the microscopic tunability and robust chemical control over the DPN architecture required for biomedical and biotechnological applications.

Efforts to construct aqueous DPNs have increasingly focused on utilizing preexisting biomolecular recognition motifs as cross‐linking elements. One notable biomolecular recognition motif is the protein‐ligand interaction between tetrameric avidin‐family proteins and biotin—the biotin‐avidin interaction is widely regarded as one of the strongest and most useful noncovalent interactions in biology [19, 20]. For example, streptavidin is the avidin‐family member from *Streptomyces avidinii* [21], and each streptavidin protomer binds one biotin molecule in a thermodynamically identical, noncooperative manner [22, 23] with a binding affinity nearly as strong as a covalent bond (K_D_ = K_A_^−1^ = 4×10^−14^ M) [19, 24]. This interaction is so strong that stoichiometric mixtures of biotin and streptavidin exhibit an apparent cooperativity that is only kinetic in nature, where nonequilibrium distributions of the completely unoccupied and completely bound streptavidin co‐exist for days until relaxation to the equilibrium binding distribution occurs [22, 23, 25]. Notably, there are many biotin‐derivatives that bind avidins, such as desthiobiotin (K_D_ = 10^−13^ M) [26], and the pH‐sensitive 2’‐iminobiotin which primarily binds in the nonprotonated guanidino form (K_D_ = 3×10^−11^ M) and exhibits apparent decreases in affinity with decreasing pH due to the equilibrium between protonated and nonprotonated states (K_D_ = 7×10^−7^ M at pH 7.5, and K_D_ ≈ 10^−3^ M at pH 4) [27, 28, 29]. Similarly, there are many avidins with unique biotin‐binding capabilities [30, 31], such as the streptavidin‐variant traptavidin with its 10‐fold increased biotin‐binding lifetime [32]. The existence of robust, in vitro bioconjugation chemistries (e.g., using maleimides [33], succinimidyl esters [34], or click chemistry [35]) and in vivo biotin‐conjugation methods (e.g., BirA ligase [36]) means that the biotin‐avidin interaction has seen extensive application to protein immobilization [37, 38], labeling [37, 39], purification [40], and, more recently, for biotechnology purposes [31, 41, 42].

Given the inherent multivalency and high thermodynamic stability of the biotin‐avidin interaction, it has been used to create static polymer networks [43, 44, 45, 46]. For example, the modular SpyAvidin Hubs developed by Howarth and coworkers are built upon a static polymer network formed from traptavidin and biotin‐conjugated biomolecules of interest (e.g., major histocompatibility complexes) [46]. A noteworthy exception to the static use of the biotin‐avidin interaction, however, is in protein purification. For instance, radiolabeled streptavidin can be eluted from iminobiotin‐functionalized chromatography resin by dropping the pH to 4 [28], because streptavidin binding is reversible. Similarly, in the Strep‐tag affinity chromatography strategy, a genetically encoded protein tag acts as a relatively weak affinity biotin‐mimic for reversible binding to the streptavidin‐variant Strep‐Tactin [40]. While polymer networks were not involved in these dynamic examples, we were inspired to transpose such tunable, reversible binding interactions onto an avidin‐based polymer network to develop control over the network topology and self‐assembly process.

**SCHEME 1:**
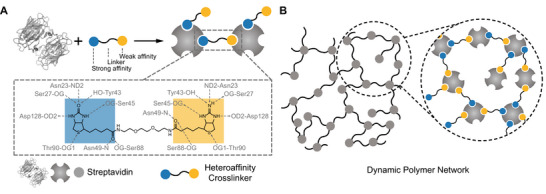
Heteroaffinity cross‐linker (HAX) forming a dynamic polymer network (DPN). (A) Schematic diagram demonstrating how the four binding pockets of streptavidin (PDB ID: 2RTN) [29] can form intermolecular interactions with the biotin‐PEG_2_‐iminobiotin (BI) HAX made of biotin (blue) and iminobiotin (yellow) as the strong and weak affinity moieties, respectively. In this example, two HAX‐bound protein “modules” are shown oligomerized by the cross‐link formed upon iminobiotin binding. (B) Schematic diagram of DPN formed by streptavidin and BI, with zoomed region showing intermolecular cross‐links.

To address the aforementioned difficulties in DPN design, we have combined the chemical diversity of the biotin‐avidin interaction with the modularity of supramolecular polymer networks to develop a novel class of biological, noncovalent, dissociative DPNs. Specifically, we created bifunctional, heteroaffinity cross‐linkers (HAX) where the two functional moieties are both biotin‐derivatives but with different affinities for avidin‐family proteins (Scheme 1). For example, with a polyethylene glycol (PEG) linker, biotin‐PEG_2_‐iminobiotin (BI) is a HAX in which both the biotin and iminobiotin functional moieties can bind streptavidin, however, the affinity of that interaction varies by ∼10^7^ at neutral pH [28, 29]. Thus, while the biotin moiety of a BI HAX binds streptavidin in an effectively irreversible manner, the iminobiotin moiety can then dynamically bind to any neighboring streptavidin that has at least one unoccupied binding site. The difference in HAX moiety affinity enables dynamic cross‐linking along with stable network formation without the need to incorporate an orthogonal chemistry, nor genetically engineer an avidin‐family protein.

To demonstrate these effects, we first show that HAX‐induced protein oligomerization creates DPNs that exhibit reversible and chemically tunable self‐assembly behavior. We then create HAX‐bound protein “modules” with monodisperse HAX valencies to enable the construction of unique, nonequilibrium DPN architectures from stoichiometrically identical components—highlighting that both equilibrium thermodynamics and kinetic programmability can be used to design polymer networks. Finally, we develop a stimuli‐responsive DPN that can be reversibly assembled and dissembled by changing the environmental pH; this DPN is capable of sequestering biotinylated molecules (e.g., small‐molecule drugs or antibody biologics) and being spatially transported using a magnetic field to a low pH region where it can release its molecular payload. Altogether, the dynamic, chemically tunable network architectures of these HAX‐based DPNs expand the functional scope of biological supramolecular chemistry and provide a foundation for the development of next‐generation, stimuli‐responsive biomaterials.

## 2 Results and Discussion

### 2.1 Monitoring HAX‐Induced Oligomerization

To create a biologically compatible DPN, we designed heterobifunctional cross‐linkers that would create transient cross‐links between streptavidin proteins (Scheme 1). While both functional moieties of the cross‐linker are biotin‐derivatives, they have significantly different affinities for streptavidin—the term HAX highlights this difference in affinity (Scheme 1A). Specifically, we made two different HAX: BI and biotin‐PEG_2_‐desthiobiotin (BD). Additionally, we made a homobifunctional cross‐linker for control experiments: biotin‐PEG_2_‐biotin (BB). The choice of a relatively short PEG_2_ linker was made to minimize intra‐streptavidin cross‐links that might inhibit DPN formation [43]. After one‐pot, 10 milligram‐scale carbodiimide‐activated syntheses of BI, BD, and BB, hydrophobic interaction chromatography (HIC)‐based purification yielded high‐purity HAX as assessed by nuclear magnetic resonance (NMR) spectroscopy and mass spectrometry (see Supporting Information and Figures S1–S8).

**FIGURE 1:**
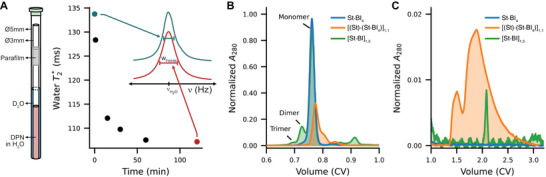
Heteroaffinity cross‐linker (HAX)‐induced streptavidin (St) oligomerization. (A) Time course of water $T_{2}^{*}$ during the assembly of a [(St)·(St·BI_4_)]_1,1_ dynamic polymer network (DPN) from the biotin‐PEG_2_‐iminobiotin (BI) HAX and St. The inset shows water peaks in ^1^H NMR spectra acquired before mixing (blue) and 120 min after mixing (red). A schematic diagram of the tube‐in‐tube NMR experiment is shown on the left. (B) A_280_ chromatogram during SEC elution for a streptavidin module (blue), a modular DPN (orange), and a nonmodular mixture of streptavidin and BI HAX (green). Curves are normalized by total area across the chromatogram. (C) Zoomed in view of chromatograms showing 49.7% of the DPN sample (orange) exhibits a negative shift in SEC, indicating complex interactions with the column resin during isocratic elution.

To test the ability of HAX to cross‐link streptavidin proteins, we sought a highly sensitive, time‐resolved method to monitor cross‐link formation in low‐concentration, aqueous solutions. In the work of Taraban and coworkers, they demonstrated that the transverse relaxation time, T_2_, of water protons in ^1^H NMR spectroscopy is an indirect reporter of the shear modulus of a DPN [47, 48]. One possible mechanism for this effect is relaxation dispersion caused by heterogeneity in the local water proton environment (e.g., exchanged onto a protein backbone in a large polymer network vs. free in the bulk), while another involves larger networks sampling more magnetic field inhomogeneities during the measurement period. Regardless of which is the dominant mechanism, the T_2_ value of proteinaceous water reports on protein aggregation [48] and can thus be used to monitor cross‐linking during DPN formation. To enable more rapid, time‐dependent measurements, we chose to measure the apparent transverse relaxation time, $T_{2}^{*}$, of water, where $1/{\;T}_{2}^{*}=1/T_{2}+1/T_{2}^{†}$ and $T_{2}^{†}$ accounts for inhomogeneous relaxation due to a nonuniform magnetic field [49]. Assuming that contributions from magnetic field nonuniformity are time‐independent during each experiment, then $T_{2}^{*}$ is a reasonable proxy for T_2_ that can be easily measured for water as $T_{2}^{*}={\left(\pi w_{FWHM}\right)}^{-1}\;$, where *w_FWHM_* is the full‐width half‐max of the water proton resonance peak in a ^1^H NMR spectrum [49]. To ensure magnetic field stability during these measurements, the aqueous DPN sample was placed in a Ø3mm NMR tube inside a Ø5mm NMR tube containing D_2_O as a deuterium source for field locking (Figure 1A). Using this approach, we found that water $T_{2}^{*}$ in the presence of agarose hydrogels decreases with agarose concentration and is stable over long periods of time, validating that water $T_{2}^{*}$ can be used as a measure of polymer network formation (Figure S9). Additionally, water $T_{2}^{*}$ was independent of streptavidin concentration in the absence of HAX (Figure S10), however it did exhibit temperature and pH dependence (Figures S11, S12). As such, all experiments were performed using a variable temperature NMR module set at 25 °C and with buffered solutions.

To assess HAX‐induced oligomerization of streptavidin, we saturated 1 mg/mL (∼19 µ*M*) streptavidin with 20‐fold molar excess of the BI HAX to create the monodisperse St·BI_4_ “module,” where St stands for streptavidin, and the center dot denotes a bound complex with four BI HAX. Given that the median lifetime of a fully biotin‐saturated streptavidin at room temperature is either 8 [23] or 18 h [50], and that the difference in binding affinity, taken together with diffusion‐limited association, suggests that the rate of iminobiotin dissociation is likely orders of magnitude faster than biotin dissociation, the four protomers of each streptavidin tetramer should be bound to the strong‐affinity biotin moiety of the BI HAX after overnight incubation. Thus, the St·BI_4_ module presented four iminobiotin moieties for inter‐streptavidin cross‐linking, however, there were no other streptavidin present with vacant binding sites for the iminobiotin to bind, so no DPN formation occurred yet.

To induce oligomerization, we mixed equimolar amounts of 1 St + 1 St·BI_4_ at a concentration of 1 mg/mL, which we expected would ultimately form a [St·BI]_1,2_ DPN once equilibrium was reached. In this notation, the square brackets denote a DPN with a stochastic, polydisperse HAX valency, and the subscripts denote the respective stoichiometries (i.e., each streptavidin binds two molecules of BI on average). Before mixing the modules, the water $T_{2}^{*}$ of the buffer alone was 135 ms, and following initiation of the cross‐linking reaction, that value decreased exponentially to ∼105 ms over the course of about 2h (Figure 1A). This decrease in water $T_{2}^{*}$ reflects the oligomerization of streptavidin in the nascent [St·BI]_1,2_ DPN network architecture. Notably, the temperature dependence of the water $T_{2}^{*}$ for streptavidin (∼1.41 ms/°C) was notably sharper than that for the [St·BI]_1,2_ DPN (∼0.70 ms/°C), suggesting that HAX‐based cross‐linking yields a thermodynamically distinct material (Figure S12).

As HAX cross‐linking induced streptavidin oligomerization, we also sought to quantify the size of the resulting oligomers. With fast protein liquid chromatography (FPLC), size‐exclusion chromatography (SEC) can separate proteins based on their hydrodynamic radii, such that increasing molecular size results in decreased on‐column retention times and elution at lower column volumes (CV). Thus, in a chromatogram of the absorption at 280 nm (A_280_), which primarily tracks the aromatic amino acids of streptavidin, we expected to observe peaks corresponding to streptavidin oligomers eluting prior to pure streptavidin. Previously, SEC has been used to quantify the size of short, linear oligomers of avidin [43] and streptavidin [44]. On a Superose 6 Increase 10/300 GL column (Cytiva), streptavidin alone eluted as a sharp peak at 0.77 CV (Figure S13), while the saturated St·BI_4_ module eluted as a slightly broader peak at 0.76 CV (Figure 1B). By mixing fivefold molar excess BI HAX with streptavidin and quickly loading it onto an SEC column, we observed peaks at 0.73 CV and 0.69 CV that correspond to streptavidin dimers and trimers, respectively, as well as a peak at 0.91 CV that corresponds to excess, unbound HAX (Figure 1B); in this case, while the BI HAX cross‐linking clearly induced stable streptavidin oligomerization, the slight excess of five BI HAX over the four binding sites per streptavidin inhibited extensive cross‐linking and limited the oligomerization to dimers and trimers rather than creating large assemblies that would elute at the column void volume; observing such cross‐linker concentration‐dependent inhibition *via* SEC is consistent with previously reported linear oligomers of streptavidin [44].

Given this insight, it was surprising that decreasing the HAX:streptavidin ratio from 5:1 to 2:1 resulted in no peaks in the A_280_ chromatogram eluting before the streptavidin monomer peak at 0.77 CV (Figure 1B). For this mixture of 1 St + 1 St·BI_4_, the area of the monomer peak was significantly reduced. Instead, the A_280_ chromatogram exhibited a shallow but very broad peak spanning ∼1.4‐2.5 CV post‐injection that contained 49.7% of the total A_280_ chromatogram area (Figure 1C). The elution of half the cross‐linked sample at volumes greater than 1 CV corresponds to a seemingly unphysical situation in which those molecular species appear to have a negative molecular mass. However, highly branched polymers are known to exhibit delayed elution in SEC, and the induced retardation is thought to be caused by entanglement of the macromolecule with the column packing material [51]. This suggests that the HAX induced streptavidin to form a large, branched, supramolecular assembly capable of interacting with the SEC resin on a macroscopic level—something not seen in previous SEC of linear avidin [43] or streptavidin [44] oligomers. Together with the water $T_{2}^{*}$ measurements, this result confirms that not only can HAX induce streptavidin oligomerization, but that the resulting clusters are macroscopic DPNs.

### 2.2 Molecular Mechanism of DPN Formation

**FIGURE 2:**
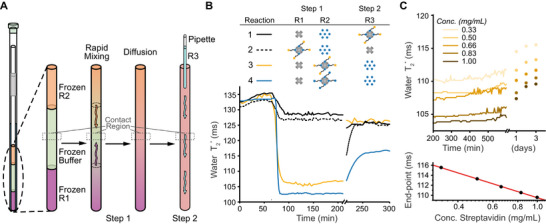
Cryo‐synchronized quenching of DPN formation. (A) Cartoon schematic of cyro‐synchronized, multistep DPN assembly experiment in an NMR tube. In the second step, a reactant R3 is added by a capillary pipette. (B) Time‐resolved measurements of water $T_{2}^{*}$ for cryo‐synchronized reactions shown in the top panel. The NMR tube is physically removed from the spectrometer at 210 min to inject reactant R3. (C) The concentration dependence of DPN formation for 1 St + 1 St·BI_4_ cryo‐synchronized reaction mixtures (top), and a plot of the final water $T_{2}^{*}$ value after three days versus streptavidin concentration (bottom). Data prior to mixing at ∼200 min has been removed here for clarity, but is provided in Figure S17.

We hypothesized that the process of HAX‐induced streptavidin oligomerization during DPN formation is a dissociative mechanism primarily driven by the weak‐affinity HAX moiety forming transient cross‐links while the strong‐affinity HAX moiety remains stably bound to streptavidin. To verify this, we performed a set of sequential quenching reactions in which mixing of the reactants was cryo‐synchronized to enable observation of the entire reaction in real‐time *via* water $T_{2}^{*}$ measurements (Figure 2A) (see Supporting Information). In these cryo‐synchronized reactions, the NMR receiver coil was positioned coincident with a frozen region of buffer that separated two reactants. Upon melting at 25 °C in a variable‐temperature NMR spectrometer, convection currents and escaping gas bubbles rapidly deliver some of the reactants to a thin “contact region” much faster than by diffusion alone (n.b., this effect can be observed by eye using food dyes; see Figure S14). Only the molecular species developing in the contact region then affect the water molecules that are measured. Importantly, not all the reactants are immediately transported to the contact region. Instead, reactants continue to slowly diffuse into the contact region, allowing for a steady‐state delivery of molecular species to be achieved. While differences in diffusion coefficients and uncertainty in the exact amount of each molecular species delivered to the contact region during rapid mixing obscure the precise reaction stoichiometries, cryo‐synchronized mixing enables assembly reactions to be observed in real‐time, and after the initial reaction, additional reactants can be delivered by pipette throughout the NMR tube (Figure 2A).

To demonstrate that DPN formation is dependent upon the presence of unoccupied streptavidin binding sites, we sought to quench HAX‐based cross‐linking by outcompeting cross‐links with excess biotin, B (Figure S15) (Reaction 1; Figures 2B, S16). More specifically, combining streptavidin with biotin to form the inert St·B_4_ would effectively inhibit any future cross‐linking to those biotin‐saturated streptavidin. After cryo‐synchronized mixing of 1 St + 20 B, we observed a stable water $T_{2}^{*}$ = 128 ms, which is consistent with the value for streptavidin at 25 °C in the absence of any network formation (c.f., Figures 1A, S10). Subsequent addition of one equivalent of St·BI_4_ as a third reactant resulted in a negligible drop to a stable water $T_{2}^{*}$ = 126 ms and showed no signs of a large drop in water $T_{2}^{*}$ that corresponds to HAX‐induced oligomerization (Figure 2B). For reference, 0.01% (w/v) agarose in water is approximately 100‐fold less concentrated than used as a hydrogel for gel electrophoresis, and only exhibits water $T_{2}^{*}$ = 124 ms (Figure S9). Altogether, this suggests that the excess biotin saturated all the streptavidin binding sites in the contact region, and thus effectively quenched any potential cross‐linking with St·BI_4_ that would have led to DPN formation.

Reasoning that the requirement of unoccupied streptavidin binding sites for DPN formation should be independent of the order of addition, we attempted to quench the cross‐linking behavior in the opposite order (Reaction 2; Figure 2B). Cryo‐synchronized mixing of 1 St·BI_4_ + 20 B led to a stable water $T_{2}^{*}$ = 127 ms, which is consistent with the presence of St·BI_4_ unaffected by biotin. As expected, the subsequent addition of one equivalent of streptavidin ultimately resulted in a water $T_{2}^{*}$ = 126 ms, which is the same value obtained at the end of Reaction 1. Thus, both orders of addition successfully quenched of HAX‐based cross‐linking and equivalently inhibited DPN formation. Surprisingly, however, the addition of one equivalent of streptavidin to the contact region resulted in a significant relaxation from an initial water $T_{2}^{*}\approx$ 115 ms to the final value of 126 ms over the course of ∼20 min (Figure 2B). This relaxation indicates that a kinetically unstable [(St)·(St·BI_4_)]_1,1_ DPN was initially formed, and that this nascent DPN was eventually disassembled as the excess biotin ultimately outcompeted the HAX cross‐links. This transient DPN formed for Reaction 2 but not Reaction 1 because the small contact region probed by the NMR receiver was only in a steady state and did not contain the amount of biotin required to immediately quench the full amount of streptavidin delivered to the contact region. Regardless, Reactions 1 and 2 demonstrate that DPN formation requires unoccupied streptavidin binding sites for HAX‐based cross‐linking.

The relatively slow disassembly of the [(St)·(St·BI_4_)]_1,1_ DPN in Reaction 2 suggested that DPN stability is governed by the binding dynamics of the weak‐affinity HAX‐moiety. To verify this hypothesis, we compared the ability of excess biotin to quench a dynamic DPN formed from HAX‐based cross‐linking versus a static polymer network formed from bifunctional biotin cross‐linking. Cryo‐synchronized mixing of 1 St + 1 St·BI_4_ to form a dynamic [(St)·(St·BI_4_)]_1,1_ DPN resulted in a sharp decrease to a stable water $T_{2}^{*}$ = 106 ms (Reaction 3; Figure 2B), which is equivalent to an ∼0.06% agarose gel (Figure S9). Similarly, cryo‐synchronized mixing of 1 St + 1 St·BB_4_ to form a static [(St)·(St·BB_4_)]_1,1_ polymer network resulted in a sharp decrease to a stable water $T_{2}^{*}$ = 103 ms (Reaction 4; Figure 2B), which is equivalent to an ∼0.08% agarose gel (Figure S9). For both reactions, the water $T_{2}^{*}$ value was stable over the course of 2h, during which the polymer networks matured toward a [St·BI]_1,2_ DPN or a [St·BB]_1,2_ polymer network. The slightly lower value of water $T_{2}^{*}$ for the static polymer network suggests that the relatively more rapid dissociation dynamics of the weak‐affinity HAX moiety limit the average network size in a DPN.

Subsequent addition of a 20‐fold excess of biotin to the dynamic DPN sample resulted in an immediate increase in water $T_{2}^{*}$ ≈ 126 ms—indicating complete disassembly of the [(St)·(St·BI_4_)]_1,1_ DPN (Reaction 3). The extent of this disassembly suggests that iminobiotin dissociated within the ∼10 min between the addition of biotin to the NMR tube and the next water $T_{2}^{*}$ measurement, and thus that dissociation of the weak‐affinity HAX moieties involved in cross‐linking is rate‐limiting for the DPN disassembly process. In contrast, addition of 20‐fold excess biotin to the static polymer network sample was expected to yield a stable network approaching a [St·BB·B]_1,2,2_ architecture, because BB cross‐links are refractory to quenching by free biotin on this timescale due to the 8 [23] to 18 [50] hour median lifetime of St·B_4_→St·B_3_ + B at 25 °C. Surprisingly, this quenching reaction exhibited a slow ∼40‐minute relaxation to a final water $T_{2}^{*}$ = 115 ms, which is consistent with the existence of a slightly smaller, but stable, polymer network despite the presence of excess quencher. Compared to the fully quenched components in Reactions 1–3 with water $T_{2}^{*}$ ≈ 126 ms, this final water $T_{2}^{*}$ = 115 ms demonstrates that DPN stability is dominated by the dynamics of the weak‐affinity HAX moiety, and those dynamics are, at most, on the minute‐timescale for the BI HAX.

While the reversible binding of the weak‐affinity HAX moiety is a requirement for DPN formation and stability, other parameters also govern DPN properties. For instance, cryo‐synchronized mixing of 1 St + 1 St·BI_4_ at different total streptavidin concentrations ranging from 0.33 mg/mL to 1 mg/mL showed that higher concentrations yielded smaller water $T_{2}^{*}$ (Figures 2C, S17). However, water $T_{2}^{*}$ was unaffected by different concentrations of streptavidin or the BI HAX alone (Figure S10). Additionally, while the DPNs relaxed to slightly smaller average network sizes over three days of maturation, their final water $T_{2}^{*}$ value was a nonlinear function of module concentration (Figure 2C, bottom), suggesting that water $T_{2}^{*}$ quantifies the network length‐scale rather than the number of cross‐links or network volume. Altogether, this evidence demonstrates that average DPN size increases as protein module concentration increases.

### 2.3 Supramolecular Tuning of DPN Properties

The material properties of a DPN depend largely on the chemical affinity of the cross‐links [5]. For a noncooperative binding interaction like that of biotin to streptavidin [22, 23], the affinity of the cross‐linking reaction for HAX‐based DPNs is governed by the thermodynamics of the bimolecular binding equilibrium *W+P⇌W·P*, where *W* is the weak‐affinity HAX moiety and *P* is the protein binding pocket. Thus, the intermolecular interactions between *W* and *P* that enthalpically define the binding energy landscape can be modified to chemically tune DPN properties. To explore this capability, we prepared several valence‐saturated modules from different combinations of *W* and *P* to assess the resulting DPN differences. Toward this goal, we expressed and purified streptavidin (St’) and the streptavidin‐variant traptavidin (Tr’) on the milligram scale using immobilized metal affinity chromatography (IMAC) (Figure S18). The oligomeric state of both was ∼95% tetrameric (Figure S19), and ∼95% of those tetramers were able to bind four biotin molecules (Figure S20) as verified by native electrospray ionization mass spectrometry (see Supporting Information). Six different valence‐saturated modules were then prepared (St’·BI_4_, St’·BD_4_, St’·BB_4_, Tr’·BI_4_, Tr’·BD_4_, and Tr’·BB_4_) by saturating the avidin‐family variant with 20‐fold excess HAX, letting the mixture equilibrate overnight, and then removing any unbound HAX by ultrafiltration.

**FIGURE 3:**
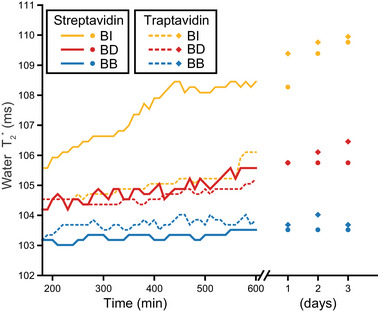
Effect of weak‐affinity HAX moiety and avidin‐variant identities on DPN assembly. Cryo‐synchronized water $T_{2}^{*}$ measurement in the presence of DPNs formed from streptavidin (solid) or traptavidin (dashed) with different HAX: biotin‐PEG_2_‐iminobiotin (BI; yellow), biotin‐PEG_2_‐desthiobiotin (BD; red), or biotin‐PEG_2_‐biotin (BB; blue). Data prior to contacting at ∼200 min has been removed here for clarity, but is provided in Figure S21.

DPNs were formed using these modules through cryo‐synchronized mixing of the valence‐saturated module in the top region with an equimolar amount of the apo‐variant in the bottom region (e.g., 1 St’·BI_4_ + 1 St’; Figure 2A). After fast, convection‐assisted mixing, all six reactions formed DPNs with low water $T_{2}^{*}$ values of approximately 105 ms (Figures 3, S21). The exact water $T_{2}^{*}$ values were inversely related to the affinity of the weak‐affinity HAX moiety (*i.e*., BB < BD < BI), suggesting that the DPN size was largely determined by the chemical properties of the HAX. After three days, the streptavidin‐based DPNs exhibited slightly lower water $T_{2}^{*}$ values than the traptavidin‐based DPNs, which may mean that streptavidin yields slightly larger networks than traptavidin; however, it could simply reflect small differences in activity, concentration, and/or tetramer stability of the IMAC‐purified St’ versus Tr’. Regardless, this experiment further confirms that DPN stability is governed by the binding dynamics of the weak‐affinity HAX‐moiety.

Interestingly, while both DPNs made with the BI HAX ultimately equilibrated to DPNs with comparable water $T_{2}^{*}$, the relaxation pathways were significantly different (Figure 3, yellow lines). This suggests that the self‐assembly reaction of the DPN proceeded by different pathways, with the St’·BI_4_‐based DPN initially yielding smaller network sizes and more rapidly approaching equilibrium than Tr’·BI_4_‐based DPN. Most likely, the initial DPN topology formed upon rapid, cryo‐synchronized mixing is a kinetically trapped, nonequilibrium structure, and relaxation to the equilibrium DPN topology is dominated by the HAX dissociation dynamics. Thus, both the self‐assembly pathway and the equilibrium macroscopic properties of the DPN can be tailored by customizing the microscopic details of the HAX cross‐linking reaction. As such, the development of novel HAX (*e.g*., tri‐functional HAX to alter branching) presents a powerful method to access unique network topologies and dynamics.

### 2.4 Modular Self‐assembly of DPNs

Since HAX‐based DPNs with unique, nonequilibrium topologies can be created from chemically distinct modules (Figure 3), we hypothesized that assembling different modules together would enable a powerful, modular approach to assembling unique, nonequilibrium DPN architectures. For example, the stoichiometrically identical reactions of 1 St + 1 St·BI_4_ and 1 St·BI_1_ + 1 St·BI_3_ would both assemble DPNs that have an average of two BI molecules per streptavidin, but their initial nonequilibrium connectivity would be the topologically distinct networks [(St)·(St·BI_4_)]_1,1_ and [(St·BI_1_)·(St·BI_3_)]_1,1_, respectively. Given sufficient time for the strong‐affinity HAX moiety to dissociate from the modules and redistribute to other streptavidin, however, both of those non‐equilibrium DPNs will ultimately equilibrate to a common [St·BI]_1,2_ DPN architecture over the course of days.

**FIGURE 4:**
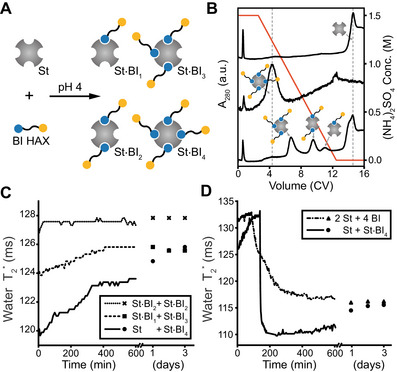
Modular self‐assembly of DPNs. (A) Schematic of different modules created from streptavidin (St) and biotin‐PEG_2_‐iminobiotin (BI) heteroaffinity cross‐linker (HAX). (B) A_280_ chromatograms of module purification using HIC. Module formation reactions being purified: 1 St (top), 1 St + 4 BI (middle), and 1 St + 1.5 BI (bottom). (C) Monitoring water $T_{2}^{*}$ during modular DPN formation from stoichiometrically identical mixtures of monodisperse protein modules. (D) Comparison of modular versus nonmodular DPN formation under cryo‐synchronized conditions with mixing occurring at ∼100‐150 min.

To test this hypothesis, we developed a method to purify monodisperse modules using HIC on an FPLC system, which allowed tracking protein elution via A_280_ (Figure 4A,B). Many groups have employed FPLC systems to purify differentially valent streptavidin [22, 52, 53]. Here, sub‐stoichiometric amounts of the BI HAX were mixed with streptavidin at a pH of 4 to avoid oligomerization. The mixture was then applied to a HIC column at high ammonium sulfate concentrations, and gradient elution fractionation to an ammonium sulfate concentration of zero separated differentially valent modules based on the number of HAX molecules bound (Figure 4B; see Supporting Information). Notably, due to the statistical binding distribution of biotin to streptavidin [22, 23] and the use of nonequilibrium biotin binding conditions in our preparation, the yield of St·BI_1_, St·BI_2_, and St·BI_3_ was relatively low compared to St·BI_4_, which can be easily made by saturating streptavidin with excess BI. Because the yield of St·BI_1_ was particularly low, we performed three modular self‐assembly reactions by mixing 1 St + 1 St·BI_4_, 1 St·BI_1_ + 1 St·BI_3_, and 1 St·BI_2_ + 1 St·BI_2_ at a low concentration of 0.1 mg/mL streptavidin and a pH of 7.2, where iminobiotin binding was not inhibited. Despite being stoichiometrically identical, the self‐assembly process differed significantly depending on the module identity (Figures 4C, S22). Based on water $T_{2}^{*}$ measurements, the initial nonequilibrium architecture of these DPNs yielded average DPN sizes in the following order: [(St)·(St·BI_4_)]_1,1_> [(St·BI_1_)·(St·BI_3_)]_1,1_> [(St·BI_2_)]_1_. While the [(St·BI_2_)]_1_ DPN began closer to the equilibrium valency, the [(St)·(St·BI_4_)]_1,1_ and [(St·BI_1_)·(St·BI_3_)]_1,1_ DPNs exhibited relaxations over the course of a day, presumably toward the equilibrium architecture of the [St·BI]_1,2_ DPN. Because the [(St)·(St·BI_4_)]_1,1_ DPN exhibited the most significant relaxation (Figure 4C), this experiment suggests that the further a protein module is from its equilibrium valency, the further the DPN architecture will be from its equilibrium topology, and that the relaxation is dominated by the hours‐long process of biotin dissociation which slowly increases the dispersity of the modules.

Finally, we compared the difference between the self‐assembly of stoichiometrically identical DPNs using modular versus nonmodular assembly mechanisms (Figure 4D). In the nonmodular case, the cryo‐synchronized reaction of 1 St + 2 BI yielded a relatively slow exponential decay to a steady‐state on the timescale of hours, and eventually achieved a final water $T_{2}^{*}$ = 117 ms representing the final, equilibrium [St·BI]_1,2_ DPN architecture. This behavior significantly contrasts with the modular formation of a DPN from the reaction of 1 St + 1 St·BI_4_, which instead yielded biphasic assembly dynamics (Figure 4D). Cryo‐synchronized mixing quickly resulted in relatively large DPNs forming in the contact region that had water $T_{2}^{*}$ = 110 ms, which is equivalent to ∼0.05% agarose gels (Figure S9). However, over the next three days, the DPN exhibited slow relaxation to the same equilibrium water $T_{2}^{*}$ = 117 ms that was achieved with nonmodular assembly. This biphasic assembly behavior confirms that modular assembly of DPNs from HAX‐based modules can access unique, nonequilibrium DPN architectures, and demonstrates how microscopic customization of the network topology enables macroscopic tuning of the DPN material properties.

### 2.5 Environmentally Responsive DPNs for Delivering Molecules to Low pH Regions

**FIGURE 5:**
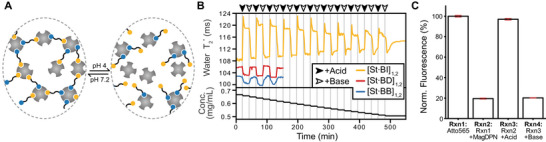
Cycling of a pH‐responsive DPN. (A) Cartoon schematic of pH cycling reaction for a BI‐based DPN. (B) Water $T_{2}^{*}$ measurement of DPN size with alternating addition of acid (black arrow) and base (white arrow) (top). Each addition dilutes DPN concentration (bottom). A well‐equilibrated [St·BI]_1,2_ DPN underwent 11 acid‐base addition cycles (yellow), where acid addition increased $T_{2}^{*}$ due to DPN disassembly at low pH. Three acid‐base addition cycles for a [St·BD]_1,2_ DPN (red) and for a static [St·BB]_1,2_ polymer network (blue) show the opposite behavior. (C) Fluorescence intensities for sequential measurements of a pH‐responsive [St·BI]_1,2_ DPN with integrated magnetic bead (MagDPN) demonstrating spatial control over pH induced release and capture of biotinylated Atto565 fluorophore. Measurements are made during magnetic pulldown, and intensities are normalized to the value for Atto565 alone. Error bars are the standard deviation of N = 3 measurements.

Because streptavidin primarily binds the nonprotonated form of 2’‐iminobiotion [29], cross‐links created by a BI HAX can be regulated by pH changes. More specifically, a DPN created with BI HAX should be stable at high pH and disassemble if the pH decreases (Figure 5A). To demonstrate this pH‐based DPN stimulus response, we monitored the water $T_{2}^{*}$ of a [St·BI]_1,2_ DPN at 1 mg/mL that had been incubated for 3 days at room temperature in phosphate‐buffered saline with a pH of 7.2. Consistent with the previous results (c.f., Figure 2C), this DPN exhibited a relatively small value of water $T_{2}^{*}$= 108 ms (Figure 5B). We then alternated adding ∼4 µL of 1 M hydrochloric acid or 1 M sodium hydroxide every 20 min to change the pH of the solution between 4.0 and 7.2 (Figures S23, S24). Through 11 cycles of acidification and neutralization of the solution, the water $T_{2}^{*}$ value instantaneously increased, then decreased by ∼15 ms, respectively, meaning that the DPN was systematically disassembled and then reassembled each cycle. In contrast, pH cycling of a [St·BD]_1,2_ DPN, where the desthiobiotin HAX moiety is not expected to exhibit a significant pH dependence like iminobiotin does, exhibited a small but opposite trend wherein acidification instead decreased $T_{2}^{*}$ (Figure 5B). The same effect was observed for a static [St·BB]_1,2_ polymer network. This effect is explained by the decrease in water $T_{2}^{*}$ caused by acidification of streptavidin solutions (Figure S11) and suggests that the disassembly efficiency of the [St·BI]_1,2_ DPN is even more pronounced than is apparent.

Interestingly, the efficiency of [St·BI]_1,2_ DPN assembly and disassembly both appeared to decrease with successive pH cycling. In part, this is due to the dilution of the DPN with each addition of acid and base (c.f., Figures 2C  and 5B, bottom), however, it likely also reflects a reduction in streptavidin stability upon addition of small amounts of acid and base, which may remain locally concentrated until sufficient mixing occurs and thus promote denaturation. Regardless, this experiment demonstrates significant, prolonged cycling of DPN assembly and disassembly in response to pH changes, and this pH responsiveness is imbued into the DPN exclusively by the chemical properties of the weak‐affinity HAX moiety.

Finally, we sought to exploit the utility of a pH‐responsive DPN to deliver molecules of interest to areas of low pH, such as a tumor microenvironment. To demonstrate this concept, we added sub‐stoichiometric amounts of a biotin‐conjugated Atto565 fluorophore (Figure S25) as a “cargo” molecule into a [St·BI]_1,2_ DPN. Specifically, the reaction 1 Atto565‐biotin + 100 [St·BI]_1,2_ yielded a DPN we call [St·BI]*_1,2,_ in which the cargo molecule is present at a low concentration to avoid significantly inhibiting the cross‐linking reaction that imbues stability to the DPN. We then sought to create a targeted delivery mechanism by incorporating magnetic nanoparticles into the DPN [12]. To do so, the [St·BI]*_1,2_ DPN was attached to commercially available magnetic‐core agarose beads that had been covalently functionalized with streptavidin, so that the DPN could be spatially controlled with an external magnetic field. As quantified by the amount of fluorescence intensity measured in a cuvette during magnetic pulldown (Figure S26), we found that we could spatially sequester 80.1% of the cargo molecule in the DPN and then release 94.0% of those sequestered cargo molecules from the DPN by acidification of the solution at a pH of 4 (Figure 5C). These delivery abilities were dependent upon the cross‐linking induced by the BI HAX (Figure S27). After release, the cargo molecules were still bound to individual modules, however, DPN disassembly decreased the local concentration of streptavidin such that the biotinylated cargo molecule would be free of exogenous components upon dissociating from that module. Finally, by neutralizing the solution back to a pH of 7.2, the DPN was reformed and was able to sequester 79.7% of the cargo molecules—99.5% as effective as the initial sequestration (Figure 5C). Thus, this magnetic nanoparticle‐bound DPN is a hybrid material that can be used as a molecular delivery or sequestration system for any biotinylated molecule (e.g., biotin‐conjugated antibodies), and one that can be macroscopically delivered by magnetic control to a region of low pH for cargo molecule release.

## 3 Conclusion

We have developed a unique set of bifunctional cross‐linkers called HAX to enable modular DPN formation from biologically compatible, aqueous components. By chemically tuning the affinity of the weak‐affinity HAX‐moiety, we were able to control the assembly kinetics, environmental response, and macroscopic behavior of the resulting DPNs. Exploiting a pH‐dependent weak‐affinity HAX moiety, we were able to engineer a DPN from streptavidin with pH‐triggered disassembly and reformation dynamics, and demonstrate its ability to capture and release biotinylated cargo molecules while being spatially controlled by magnets. As such, HAX‐based DPNs hold promise as a drug delivery platform. More importantly, by creating a series of monodisperse protein modules, we demonstrated that HAX enables the modular assembly of unique nonequilibrium DPN architectures from stoichiometrically identical components. Leveraging this kinetic control over the network topology, along with the chemical tunability and stimuli responsiveness of HAX‐based DPNs, will enable the development of many new and dynamic biocompatible materials.

## Conflicts of Interest

The authors declare no conflicts of interest.

## Supporting information




**Supporting File 1**: The authors have cited additional references within the Supporting Information [1–5].

## Data Availability

The data that support the findings of this study are available in the supplementary material of this article.
