# Supplementary material for: Modular Assembly of Dynamic Polymer Networks From Heteroaffinity Cross‐Links to Multivalent Proteins
Source: Angew Chem Int Ed Engl. 2026 Feb 5;65(17):e26058. doi: 10.1002/anie.202526058 (PMC13098475; doi:10.1002/anie.202526058)
Supplement: Supplementary file 1 — Supporting File 1: The authors have cited additional references within the Supporting Information [1–5]. [file ANIE-65-e26058-s001.docx]

***SUPPORTING INFORMATION***

**Modular assembly of dynamic polymer networks from heteroaffinity cross-links to multivalent proteins**

Tianyue Dai^a†^, Yuntao Qiu^a†‡^, Katherine M. Leon Hernandez^a^, Wesley Chan^a^, Katrina Mejia^a^, Natalia Nemeria^a^, Colin D. Kinz-Thompson^a*^

a. Department of Chemistry, Rutgers University–Newark, Newark, NJ 07102

† These authors contributed equally to this work

‡ Current Address: The Advanced Science Research Center, Graduate Center of the City University of New York, New York, NY 10031, USA, and the Ph.D. program in Biochemistry, Graduate Center of the City University of New York, New York, NY 10016, USA.

* Email: colin.kinzthompson@rutgers.edu

***TABLE OF CONTENTS***

***Supporting Methods***

1. Heteroaffinity cross-linker synthesis
2. Protein expression and purification
3. Native electrospray ionization mass spectrometry
4. Preparation of saturated, HAX-bound protein modules
5. Cryo-synchronized reactions
6. Water NMR and data analysis
7. Experimental details
   1. HAX-induced DPN formation
   2. Order-of-addition quenching experiments
   3. Concentration-dependence of DPN formation
   4. Chemical tunability of DPN behavior
   5. Modular assembly of stoichiometrically identical DPNs
   6. Modular versus non-modular DPN formation
   7. Cycling of a pH-responsive DPN
   8. Spatially controlled delivery of biotinylated molecules

***Supporting Figures***

1. ^1^H NMR spectrum of BI
2. ^13^C NMR spectrum of BI
3. HSQC spectrum of BI
4. ESI MS spectrum of BI
5. ^1^H NMR spectrum of BD
6. ESI MS spectrum of BD
7. ^1^H NMR spectrum of BB
8. ESI MS spectrum of BB
9. Effect of agarose polymer networks on water $T_{2}^{*}$
10. Concentration-dependence of water $T_{2}^{*}$ for streptavidin and BI HAX
11. pH-dependence of water $T_{2}^{*}$ in the presence of streptavidin
12. Temperature-dependence of water $T_{2}^{*}$ in the presence of a DPN
13. Full SEC chromatograms of streptavidin DPNs
14. Demonstration of cryo-synchronized mixing of food dyes
15. ESI MS spectrum of biotin
16. Cryo-synchronized quenching experiment replication
17. Concentration-dependence of [St·BI]_1,2_ DPN formation time course
18. Electrophoretic analysis of non-denaturing purification
19. Native ESI MS analysis of oligomeric state
20. Native ESI MS analysis of biotin-binding activity
21. Time course of DPN formation from different protein modules
22. Modular assembly experiment replication
23. Schematic diagram of cycling of pH-sensitive DPN experiment
24. pH cycling experiment replication
25. ESI MS spectrum Atto 565-biotin
26. Schematic diagram of controlled molecular delivery experiment
27. Control experiments for controlled molecular delivery

**Supporting References**

***Supporting Methods***

**1. Heteroaffinity cross-linker synthesis**

All biotin analogs (2’-iminobiotin, biotin and d-desthiobiotin), ammonium sulfate ((NH_4_)_2_SO_4_), sodium hydroxide (NaOH), 4-dimethylaminopyridine (DMAP), sodium chloride (NaCl), ethanol (EtOH; 200 proof), and the methanol (>99.9%) used as high resolution electrospray ionization mass spectrometry (HR ESI MS) solvent were purchased from Millipore-Sigma. Triethanolamine (TEA) and dimethyl sulfoxide (DMSO) were purchased from Oakwood Chemical. 1-ethyl-3-(3-dimethylaminopropyl carbodiimide hydrochloride (EDC·HCl) and EZ-Link Amine-PEG_2_-Biotin were purchased from ThermoFisher Scientific. All compounds were used without further purification. Buffers were prepared using ultrapure-grade water (18.2 MΩ/cm) prepared with a Barnstead Genpure II water purification system. Deuterium oxide (D_2_O; 99.8%) was purchased from Acros Organics.

Heteroaffinity cross-linker (HAX) synthesis was performed in a one-pot, EDC-activated, condensation reaction, in which 64.1 μmol of a biotin analog (15.6 mg iminobiotin, 13.8 mg desthiobiotin, or 15.7 mg biotin) was mixed with limiting EDC·HCl (53.4 μmol, 10.24 mg) and DMAP (53.4 μmol, 6.5 mg) in 2 mL anhydrous DMSO, and then 11.2 μL TEA (80.1 μmol) was added into the mixture. The mixture was stirred at room temperature overnight. The solution was dried under vacuum, and the resulting white powder was dissolved in 0.5 mL ultrapure water. This crude reaction mixture was purified using hydrophobic interaction chromatography (HIC) on an ӒKTA pure 25 M (Cytiva) fast protein liquid chromatography (FPLC) system kept at 5 °C. The aqueous mixture was supplemented with 1.5 M (NH_4_)_2_SO_4_ and loaded onto a HiTrap Phenyl HP column (Cytiva) pre-equilibrated with 1.5 M (NH_4_)_2_SO_4_ aqueous solution at a system flow rate of 2 mL/min for 25 mL. Gradient elution fractionation with a linear, 20 column volume (CV) gradient to 0 M (NH_4_)_2_SO_4_ followed by a 5 CV column wash in pure water separated the crude reaction mixture components. Bifunctional cross-linker product was monitored by absorption at 205 nm, which showed peaks eluting at ~30-45% Buffer B. The corresponding fractions were pooled and then desalted by washing in ethanol. Pure cross-linker product was then vacuum dried with a lyophilizer (Labconco) to yield a white powder, and yield was determined by mass. Final products were dissolved in phosphate buffered saline, pH 7.2 (PBS; 50 mM sodium phosphate pH 7.2, 100 mM sodium chloride) at a final concentration of 5 mM, and stored at 4 ℃ for future use.

Nuclear magnetic resonance (NMR) spectroscopy was performed with a Bruker Avance III HD 500 MHz NMR Spectrometer at 499.9 MHz (^1^H) or 125.7 MHz (^13^C) that was equipped with a Bruker Smart Variable Temperature (BSVT) system. All spectra were acquired using a 5mm BBO broadband probe with Z-gradient. ^1^H and ^13^C spectra were internally referenced to D_2_O. Spectra were analyzed using TopSpin 4.4.0 (Bruker) and Mnova 15 (Mestrelab Research) software. Abbreviations used are: s, singlet; d, doublet; t, triplet; q, quartet; m, multiplet.

HR ESI MS was performed with an Orbitrap Exploris 240 (Thermo Scientific) in positive or negative mode using the following settings: Positive Ion Spray Voltage, 3400 V; Negative Ion Spray Voltage, 2300 V; Ion Transfer Tube Temp., 320 °C; Resolution, 30000; Scan range, 100-1500; RF Lens, 120%. Samples were diluted in methanol to a concentration of 5 $\mu$M and then directly injected into the spectrometer at a flowrate of 10 μL/min.

*Biotin-PEG_2_-Iminobiotin (BI)*. ^1^H NMR (500 MHz, D_2_O): δ 4.84-4.81 (m, 1H), 4.65-4.61 (m, 2H), 4.45-4.42 (m, 1H), 3.69 (s, 4H), 3.65-3.63 (t, 4H), 3.46-3.43 (m, 1H), 3.42-3.39 (t, 4H), 3.35-3.34 (m,1H), 3.08-3.04 (dd, 1H), 3.02-2.99 (dd, 1H), 2.92-2.89 (d, 1H), 2.81-2.78 (d, 1H), 2.30-2.27 (t, 4H), 1.80-1.57 (m, 8H), 1.47-1.43 (m, 4H). ^13^C NMR (125 MHz, D_2_O): δ 176.92, 165.35, 159.63, 69.50, 68.90, 65.06, 63.34, 62.10, 60.27, 55.43, 55.23, 39.75, 39.21, 38.92, 35.94, 35.49, 27.91, 27.73, 27.63, 25.13. HR ESI MS *m/z* [M+H]^+^ calculated for C_26_H_45_N_7_O_5_S_2_ 600.29964, found 600.2966.

*Biotin-PEG_2_-Desthiobiotin (BD)*. ^1^H NMR (500 MHz, D_2_O): δ 4.7-4.68 (m, 1H), 4.51-4.49 (m, 1H), 4.01-3.96 (m, 1H), 3.88-3.83 (m, 1H), 3.76 (s, 4H), 3.72-3.69 (t, 4H), 3.48-3.46 (t, 4H), 3.43-3.39 (m, 1H), 3.09-3.06 (dd, 1H), 2.88-2.85 (d, 1H), 2.37-2.33 (m, 4H), 1.84-1.59 (m, 8H), 1.53-1.37 (m, 6H), 1.19-1.18 (d, 3H). HR ESI MS *m/z* [M+H]^+^ calculated for C_26_H_47_N_6_O_6_S 571.32723, found 571.3249; [M+Na]^+^ calculated for C_26_H_46_N_6_O_6_SNa 593.30917, found 593.3069.

*Biotin-PEG_2_-Biotin (BB)*. ^1^H NMR (500 MHz, D_2_O): δ 4.63-4.60 (m, 2H), 4.43 (m, 1H), 3.69 (s, 4H), 3.65-3.62 (t, 4H), 3.41-3.40 (t, 4H), 3.34 (m, 2H), 3.02-2.98 (dd, 2H), 2.80-2.78 (d, 2H), 2.30-2.27 (t, 4H), 1.73-1.60 (m, 8H), 1.42 (m, 4H). HR ESI MS *m/z* [M+H]^+^ calculated for C_26_H_44_N_6_O_6_S_2_ 601.28365, found 601.2814; [M+Na]^+^ calculated for C_26_H_44_N_6_O_6_S_2_ 623.26560, found 623.2635.

**2. Protein expression and purification**

Streptavidin (St’) and traptavidin (Tr’) were recombinantly expressed in *Escherichia coli* (*E. coli*), and purified from the soluble fraction rather than with the previously published, denaturing approach.^[1]^ While the protein yield of this non-denaturing approach is lower (typically ~1.5-2 mg/L of culture), it is simpler, does not require refolding and tetramerization to yield functional protein, and produces at least ~95% tetrameric protein that is ~ 95% active (see Section 3). With this purification approach, the pET21a-Streptavidin-alive or pET21a-Traptavidin expression vector was transformed into electrocompetent *E. coli* T7 Express cells (C2566H, New England Biolabs). The pET21a-Streptavidin-Alive was a gift from Alice Ting (Addgene plasmid #20860), and the pET21a-Traptavidin was a gift from Mark Howarth (Addgene plasmid #24753). Cells containing the expression vector were grown overnight in Luria Bertani medium supplemented with 50 mg/mL of ampicillin at 24 °C overnight, and used to inoculate 800 mL of LB medium containing 50 mg/mL of ampicillin in a Thomson Ultra Yield Flask at 30 °C while shaking at 250 RPM in a temperature-controlled shaking incubator. Once the culture reached an OD_600_ of 0.5–0.7, the temperature was lowered to 15 °C, and protein expression was induced by addition of 0.5 mM isopropyl $\beta$-D-1-thiogalactopyranoside (IPTG). After an overnight induction at 15 °C, cells were pelleted by centrifugation at 7,000 RPM for 5 minutes at 4 °C (JA-14 fixed angle rotor, Beckman Coulter). The resulting cell pellet was washed with 50 mM potassium phosphate buffer (pH 7.5) and 150 mM NaCl, and then stored at -20 °C until purification.

Both St’ and Tr’ were purified using immobilized metal affinity chromatography. Briefly, frozen cell pellets were placed on ice and resuspended in ice-cold lysis buffer composed of 50 mM Tris**-**HCl (pH_RT_ 7.8), 300 mM NaCl, 1 mM benzamidine**-**HCl, 10 mM imidazole and 0.5% Triton X-100. Then, phenylmethylsulfonyl fluoride was added to a final concentration of 1 mM, and the cells were treated for 20 min by lysozyme added to a final concentration of 0.6 mg/mL. Cells were then sonicated for 10 min in 20 sec “pulse on” and 20 sec “pulse off” intervals (Branson 250 Digital Sonifier Ultrasonic Cell Disruptor). The lysate was clarified by centrifugation at 14,000 RPM for 20 min at 4 °C (JA-20 fixed angle rotor, Beckman Coulter) and the resulting supernatant was bound at room temperature to a 5 mL HisTrap HP column (Cytiva) pre-equilibrated with binding buffer composed of 50 mM Tris-HCl (pH_RT_ 7.8), 300 mM NaCl, and 10 mM imidazole. After binding, the mobile phase was fractionated under isocratic elution with elution buffer composed of 50 mM Tris-HCl (pH_RT_ 7.8), 300 mM NaCl, and 300 mM imidazole. Fractions were analyzed by 8% sodium dodecyl sulfate–polyacrylamide gel electrophoresis (SDS PAGE) by mixing samples with loading buffer without boiling to avoid tetramer dissociation (Fig. S18), however because these are pseudo-denaturing conditions, interpreting the protein complex size is not straightforward and the observed oligomeric ratio is not necessarily accurately representative of the sample. Gels were stained using Coomassie stain. Pure, proteinaceous fractions were dialyzed using Spectra/Por dialysis tubing with a 12-14 kDa molecular weight cutoff (MWCO) overnight at 4 °C against 1 L of 50 mM Tris-HCl buffer (pH_RT_ 7.8) and 150 mM NaCl. Protein was concentrated and exchanged into buffer composed of 100 mM Tris-HCl (pH_RT_ 7.5), 150 mM NaCl, and 1.0 mM ethylenediaminetetraacetic acid (EDTA) by means of centrifugal filtration with a 10 kDa Amicon centrifugal filter (Millipore-Sigma). Concentration was determined using the Bradford assay. Finally, glycerol was added to 5% final concentration, and 0.2 mL aliquots were flash-frozen in liquid N_2_, and stored at -80 °C until use.

**3. Native electrospray ionization mass spectrometry**

For native ESI MS experiments, it was necessary to exchange St’ or Tr’ from the non-volatile tris-based buffer used for storage into a volatile 50–150 mM ammonium acetate solution (pH 6.8). Ammonium acetate acts as a stabilizing, background electrolyte during ESI MS, and provides maximal buffering around pH 4.75 (acetate) or 9.25 (ammonium) although it results in a neutral pH upon dissolving in water.^[2,3]^ In addition, 0.5% 3-Nitrobenzyl alcohol (*m*NBA) was employed as a supercharging reagent to shift the range of the protein charge-state distribution to better match the range of the mass spectrometer, and to increase sensitivity and suppress adducts.^[4]^ PD-10 desalting columns (Cytiva) were employed for buffer exchange, and samples were then concentrated concentration using 10 kDa MWCO Amicon centrifugal filters (Millipore Sigma). For each experiment, a concentrated stock solution of St’ (5.94 mg/mL, 106 $\mu$M) was diluted to 3.4 $\mu$M in 150 mM ammonium acetate (pH 6.8) containing 0.5% *m*NBA and different amounts of biotin within the 0.57-5.0 $\mu$M range; the final volume was 1 mL. Samples were acclimated to room temperature (20 °C), and then injected into the spectrometer for measurement. An equivalent procedure was used for Tr’.

Native ESI MS measurements were performed with an Orbitrap Exploris 240 (Thermo Scientific) in positive mode using the following settings: Positive Ion Spray Voltage, 3400 V; Sheath Gas, 10.0 Arb; Aux Gas, 5.0 Arb; Sweep Gas, 0.0 Arb; Ion Transfer Tube Temp., 320 °C; Vaporizer Temp., 0 °C; Resolution, 15000; Scan range, 1000-3500; RF Lens, 120%; AGC Target, Standard; Max Injection Time, Auto; Microscans, 10. Sample was injected through a 100 $\mu$L syringe (Hamilton) by syringe pump (Chemyx) at flow rates of 10–30 $\mu$l/min. Before performing an experiment, Carbonic Anhydrase (Millipore) was used as standard sample to ensure the spectrometer was optimally functional. Mass-to-charge ratio spectra were deconvolved into mass spectra by means of UniDec software, which uses a Bayesian deconvolution algorithm that enables fast and quantitative processing.^[5]^ Mass spectra were analyzed using custom python scripts to demonstrate that both streptavidin and traptavidin samples are ~95% tetrameric proteins (Fig. S19), and that the tetramers are ~95% active for binding biotin (Fig. S20).

**4. Preparation of saturated, HAX-bound protein modules**

To prepare the St·BI_4_ HAX-bound protein module, a 1 mg/mL (19 μM) solution of core streptavidin (S888, ThermoFisher Scientific) was made in 1 mL of 50 mM PBS buffer pH 7.2. This solution was mixed with 0.76 mL 500 μM BI HAX (380 μM, 20 equivalents). The mixture was incubated for two hours at room temperature. Separation of St·BI_4_ from unbound HAX was performed by: (*1*) centrifugal filtration using an Amicon Ultra Centrifugal Filter, 10kDa (UFC5010, Millipore-Sigma) three times centrifuging for 20 mins at 14,000g, or (*2*) size exclusion chromatography (SEC) on an FPLC system using a Superose 6 Increase 10/300 GL column (Cytiva) where the St·BI_4_ eluted at 0.78 CV, and was concentrated by ultracentrifugation using a 10 kDa MWCO Amicon centrifugal filter (Millipore Sigma) to a final concentration of 18.87 μM (1 mg/mL) and stored at -20 ℃ for future use. The St’·BI_4_, St’·BD_4_, St’·BB_4_, Tr’·BI_4_, Tr’·BD_4_, and Tr’·BB_4_ modules used in Fig. 3 (see Section 7D) were prepared in a similar manner but from St’ and Tr’ stock solutions produced as described in Section 2.

**5. Cryo-synchronized reactions**

To prepare a cryo-synchronized reaction to be monitored by water $T_{2}^{*}$ measurements, parafilm was first wrapped around a 7” tall Ø3mm NMR tube at ~5” from the bottom to ensure it would remain vertical inside an 8” tall Ø5mm NMR tube. Before placing the Ø3mm tube inside the Ø5mm tube, a frozen structure was first created by adding 100 μL of Reactant 1 (R1) and freezing the solution in a freezer at -20 ℃. To minimize melting, 100 μL of ice-cold PBS buffer pH 7.2 was added on top of the frozen R1, and the tube was immediately frozen again. Then, 100 μL of ice-cold Reactant 2 (R2) was added to the top of the frozen structure, and the tube was frozen once more. When ready to initiate the reaction, the tube was removed from the freezer, the exterior was wiped, and then quickly inserted into an Ø5mm NMR tube containing 600 μL of D_2_O, which was then placed into the temperature-controlled NMR spectrometer for water $T_{2}^{*}$ measurements as described in Section 6. Notably, mixing of R1 and R2 was not dominated by diffusion, but instead by convection currents and air bubbles during melting which rapidly transported mass to the ‘contact region’ of the NMR tube (see Fig. 2A). This effect can be clearly seen with yellow and blue food dyes as R1 and R2, respectively, at the 4- and 5-minute pictures in Fig. S14.

**6.** **Water NMR and data analysis**

A Bruker Avance III HD 500 MHz NMR spectrometer with BSVT system for temperature control was used to collect data from non-deuterated, aqueous samples in an Ø3mm NMR tube co-axially placed inside an Ø5mm NMR tube containing D_2_O (see Section 5). ^1^H NMR spectra at 499.9 MHz were collected using a 5 mm BBO broadband probe. Magnetic field stability was maintained by locking to the deuterium signal of the D_2_O surrounding the Ø3mm co-axial NMR tube containing the sample. For time courses used to monitor time-dependent dynamic polymer network (DPN) formation, IconNMR inside the TopSpin 3.6.2 software was used to automate acquisition every 1-10 min. For temperature-dependent measurements (*e.g.*, in Fig. S12), the BSVT system was used to actively heat or cool the by delivering temperature-controlled nitrogen gas, while a thermocouple in the probe provided active feedback to the BSVT system. The probe temperature was manually monitored until it stabilized at the target temperature before acquiring a ^1^H NMR spectrum.

After obtaining a ^1^H NMR spectrum of an aqueous sample, the data were imported into TopSpin 4.4.0 and the water ^1^H resonance was calibrated to 4.79 ppm. The frequencies where the lineshape of this peak dropped below half the maximum value were located, and the difference was used as the full-width half-maximum (w_FWHM_) of this peak in Hz. The water $T_{2}^{*}$ value for the solution at the time and temperature the spectrum was acquired was then calculated as $T_{2}^{*}=\left( \pi\cdot w_{FWHM} \right)^{-1}$.

**7. Experimental details**

*7A. HAX-induced DPN formation*

To collect the data used in Fig. 1A, 200 µL of 0.5 mg/mL streptavidin (S888, ThermoFisher Scientific) in PBS pH 7.2 was added to a co-axial NMR tube and a 1H NMR spectrum was acquired. To initiate DPN formation, 100 µL of 1.0 mg/mL St·BI_4_ (see Section 4) was added to the NMR tube, which was immediately placed in the NMR spectrometer and ^1^H NMR spectra were acquired at 1, 15, 30, 60, and 120 min post-initiation. Water $T_{2}^{*}$ was calculated for each spectrum as described in Section 6.

To collect the size exclusion chromatography (SEC) chromatograms shown in Fig. 1B-1C and Fig. S13, samples were loaded into a Superose 6 Increase 10/300 GL (Cytiva) SEC column with PBS buffer pH 7.2 as the mobile phase at a system flow rate of 0.5 mL/min. After 5 mL of sample application, 3 CV of isocratic elution was performed, and the absorption at 280 nm was monitored using the UV-detector of the ӒKTA pure 25 M (Cytiva) FPLC system. Samples applied were: (1) 50 µL of 0.66 mg/mL St (12.6 µM); (2) 50 µL of 0.66 mg/mL St·BI_4_ (12.6 µM); (3) 50 µL of a mixture of 0.33 mg/mL St (6.3 µM) + 0.33 mg/mL St·BI_4_ (6.3 µM) injected immediately after mixing; and (4) 50 µL of a 1:1 v:v mixture of 0.66 mg/mL St (12.6 µM) + 63 µM BI HAX injected immediately after mixing.

*7B. Order-of-addition quenching experiments*

Cryo-synchronized reactions shown in Fig. 2B were prepared as described in Section 5. After removing the NMR tube from ice and placing it in NMR spectrometer, cryo-synchronized mixing was monitored for 210 minutes by acquiring ^1^H NMR spectrum every 1-5 min; significant mixing of reactant 1 (R1) and reactant 2 (R2) began at ~75 min. After 210 minutes, the NMR tube was ejected from the NMR spectrometer, and 10 μL of reactant 3 (R3) was dispersed throughout the NMR tube by slowly dispensing it from a capillary tube in the end of a micropipette while slowly withdrawing the capillary through all three regions of the NMR tube (*c.f.*, Fig. 2A). The NMR tube was then placed back into the NMR spectrometer, and 1H NMR spectra were acquired every 5 min from 220 mins until 300 mins post-initiation. Samples and amounts for reactions 1-4 are provided in Table S1. Biotin was purchased from Sigma-Aldrich (B4639), and used without further purification; HR ESI MS *m/z* [M-H]^-^ calculated for C_10_H_15_N_2_O_3_S 243.08089, found 243.0815 (Fig. S15).

**Table S1.** Reactants used in cryo-synchronized quenching reactions.

| *Reaction* | *R1 (100 µL)* | *R2 (100 µL)* | *R3 (10 µL)* |
| --- | --- | --- | --- |
| 1 | 18.9 µM St | 377 µM biotin | 189 µM St·BI_4_ |
| 2 | 18.9 µM St·BI_4_ | 377 µM biotin | 189 µM St |
| 3 | 18.9 µM St | 18.9 µM St·BI_4_ | 3.77 mM biotin |
| 4 | 18.9 µM St | 18.9 µM St·BB_4_ | 3.77 mM biotin |

*7C. Concentration-dependence of DPN formation*

Cryo-synchronized reactions of 1 St + 1 St·BI_4_ were performed as a function of the total streptavidin concentration (Fig. 2C). In all cases, R1 was 1mg/mL of St (18.9 µM) and R2 was 1mg/mL of St·BI_4_ (18.9 µM). 300 µL DPNs with final streptavidin concentrations of 0.33 mg/mL (6.3 µM), 0.5 mg/mL (9.4 µM), 0.66 mg/mL (12.6 µM), 0.83 mg/mL (15.7 µM), 1 mg/mL (18.9 µM) were created by using 1 mg/mL concentrations of R1 and R2 with volumes each of 50 μL, 75 μL, 100 μL, 125 μL, and 150 μL, respectively, and with a corresponding buffer region volumes of 200 μL, 150 μL, 100 μL, 50 μL, and 0 μL of PBS, respectively. After inserting the cryo-synchronized reaction NMR tubes into the NMR spectrometer, ^1^H spectra were acquired every 1-10 min for 600 mins. After this period, the NMR tube was removed from the NMR spectrometer and kept at room temperature (~20 °C) until one, two, and three days had elapsed, and at each point another ^1^H NMR spectrum was acquired.

*7D. Chemical tunability of DPN behavior*

HAX-bound protein modules made of recombinantly expressed and purified streptavidin (St’) or traptavidin (Tr’) (see Section 2) were prepared as described in Section 4. These modules were used to cryo-synchronize the formation of distinct DPNs (see Section 5) and monitor the formation using water $T_{2}^{*}$ (see Section 6) as shown in Fig. 3. In all cases, R1 was 100 μL of 1 mg/mL St’ (17.7 μM) or 1 mg/mL Tr’ (17.8 μM), and the buffer region was 100 μL of PBS pH 7.2. The identity of R2 varied by experiment, but was 100 μL of 1 mg/mL of St’·BI_4_, St’·BD_4_, St’·BB_4_, Tr’·BI_4_, Tr’·BD_4_, or Tr’·BB_4_. Reactions were initiated by inserting the NMR tube in the NMR spectrometer at 25 °C, and ^1^H NMR spectra were acquired every 1-5 min for 600 mins. After this period, the NMR tube was removed from the NMR spectrometer and kept at room temperature (~20 °C) until one, two, and three days had elapsed, and at each point another ^1^H NMR spectrum was acquired. Water $T_{2}^{*}$ time courses shown in Fig. 3 are truncated to avoid the initial mixing event, but the full time courses are shown in Fig. S21.

*7E. Modular assembly of stoichiometrically identical DPNs*

Stable, differentially valent HAX-bound protein modules formed from streptavidin and the BI HAX were purified using HIC under acidic conditions on an ӒKTA pure 25 M FPLC system (Cytiva). A mixture containing significant concentrations of St·BI_1_, St·BI_2_, and St·BI_3_ was prepared by mixing 350 μL of 18.9 μM St with 200 μL of 50 μM BI HAX, which is in a 2:3 molar ratio. This mixture was made in citrate buffer pH 4.0 (50 mM sodium citrate dihydrate, 100 mM NaCl) prepared using ultrapure-grade water. The mixture was supplemented to 1.5 M (NH_4_)_2_SO_4_, and immediately injected into the FPLC system kept at 5 °C without additional incubation. The sample was loaded onto a HiTrap Phenyl HP column (Cytiva) pre-equilibrated with Buffer B (1.5 M (NH_4_)_2_SO_4_, 50 mM citric acid, 100 mM NaCl, pH 4) at a system flow rate of 3.8 mL/min for 10 mL. Gradient elution fractionation with a linear, 10 CV gradient to 100% buffer A (50 mM sodium citrate dihydrate, 100 mM NaCl, pH 4), followed by a 3 CV column wash separated the differentially valent modules (Fig. 4B). Fractions corresponding to each module were determined from the A_280_ chromatogram, pooled together, and concentrated at 5 °C using a 10 kDa MWCO Amicon centrifugal filter (Millipore Sigma).

To form stoichiometrically identical DPNs from these modules, the samples were buffer exchanged into PBS by ultracentrifugation using three washes in a 10 kDa MWCO Amicon centrifugal filter (~20 minutes each), and diluted with PBS to 4 μM with concentration determined *via* Bradford assay. During this buffer exchange time, the modules likely formed homogeneous DPNs, although the valencies of individual modules remained stable given the slow dissociation rate of biotin from streptavidin. Three un-synchronized reactions of these modules were prepared in co-axial NMR tubes as described in Section 5. In each reaction, 150 µL of 4 μM R1 was directly mixed with 150 µL of 4 μM R2, where R1 and R2 were: (1) St and St·BI_4_; (2) St·BI_1_ and St·BI_3_; and (3) St·BI_2_ and St·BI_2_. After mixing, the reaction was immediately transferred into the NMR spectrometer, and ^1^H spectra were acquired every 5 min for 600 mins. After this period, the NMR tube was removed from the NMR spectrometer and kept at room temperature (~20 °C) until one, two, and three days had elapsed, and at each point another ^1^H NMR spectrum was acquired.

*7F. Modular versus non-modular DPN formation*

The two cryo-synchronized, stoichiometrically identical reactions shown in Fig. 4D were prepared as described in Section 5. In the modular reaction, R1 was 100 µL of 1 mg/mL St (18.9 µM) and R2 was 100 µL of 1 mg/mL of St·BI_4_ (18.9 µM). In the non-modular reaction, R1 was 100 µL of 2 mg/mL St (37.7 µM) and R2 was 100 µL of 76 µM BI HAX. After transferring the NMR tube into the NMR spectrometer at 25 °C, ^1^H spectra were acquired every 2-10 min for 600 mins. After this period, the NMR tube was removed from the NMR spectrometer and kept at room temperature (~20 °C) until one, two, and three days had elapsed, and at each point another ^1^H NMR spectrum was acquired.

*7G. Cycling of a pH-responsive DPN*

In the experiment shown in Fig. 5B, well-equilibrated DPNs were prepared by incubation in Ø3mm NMR tubes at room temperature for three days. Specifically, 300 μL of a stable [St·BI]_1,2_ DPN was prepared from 18.9 μM St and 18.9 μM St·BI_4_ in PBS buffer pH 7.2. Similarly, 300 μL of a stable [St·BD]_1,2_ DPN was prepared from 18.9 μM St and 18.9 μM St·BD_4_ in PBS buffer pH 7.2, and 300 μL of a static [St·BB]_1,2_ polymer network was prepared from 18.9 μM St and 18.9 μM St·BB_4_ in PBS buffer pH 7.2. After incubation, this NMR tube was inserted co-axially into a larger Ø5mm NMR tube containing 600 μL D_2_O, and placed in the NMR spectrometer. For the next 10 min, ^1^H NMR spectra at 499.9 MHz were automatically acquired every minute using the IconNMR software in TopSpin 3.6.2. After this initial time period, the sample was removed from the NMR spectrometer and a capillary tube in a micropipette was used to deliver 4.0 μL of 1 M HCl throughout the NMR tube to drop the pH to 4, and acquisition of ^1^H NMR spectra was continued every minute for 20 more minutes. The NMR tube was removed again, and 4.0 μL of 1 M NaOH was added throughout the NMR tube to increase the pH back to 7.2, and acquisition of ^1^H NMR spectra was continued every minute for 20 more minutes. The processes of adding HCl and acquiring ^1^H NMR spectra for 20 min, then adding NaOH and acquiring ^1^H NMR spectra for 20 more minutes is referred to as a single “cycle” (see Fig. S23). In total, we performed 11 cycles for the St·BI_4_ DPN, 3 cycles for the St·BD_4_ DPN, and 3 cycles for the static St·BB_4_ polymer network (Fig. 5B), and slightly increased the volume of HCl and NaOH added each cycle to achieve the desired pH (Table S2). The experiment was also repeated for 3 cycles of the St·BI_4_ DPN (Fig. S24).

**Table S2.** Acid/base volumes for pH cycling and resulting pH.

| Cycle | Volume 1M HCl (μL) | pH After Addition^a^ | Volume 1M NaOH (μL) | pH After Addition^a^ |
| --- | --- | --- | --- | --- |
| 1 | 4.0 | 3.88 | 4.0 | 7.20 |
| 2 | 4.1 | 3.73 | 4.1 | 7.19 |
| 3 | 4.1 | 3.99 | 4.1 | 7.19 |
| 4 | 4.15 | 4.02 | 4.15 | 7.20 |
| 5 | 4.25 | 4.00 | 4.25 | 7.20 |
| 6 | 4.3 | 4.00 | 4.3 | 7.21 |
| 7 | 4.4 | 3.96 | 4.4 | 7.23 |
| 8 | 4.5 | 3.85 | 4.5 | 7.25 |
| 9 | 4.6 | 3.88 | 4.6 | 7.23 |
| 10 | 4.7 | 3.76 | 4.7 | 7.25 |
| 11 | 4.8 | 3.68 | 4.8 | 7.21 |
| ^a^ pH measured by micro pH electrode in equivalent experiment at 100x volume, *i.e.*, ~400 μL additions to 30 mL PBS buffer alone. | | | | |

*7H. Spatially controlled delivery of biotinylated molecules*

Delivery of biotinylated ‘cargo’ molecules from a DPN at low pH and their subsequent re-capture at high pH was demonstrated in Fig. 5C. The ‘cargo’ molecule was an Atto 565-biotin conjugate (Sigma-Aldrich, 92637) that was used without further purification; HR ESI MS *m/z* [M]^+^ calculated for C_46_H_57_N_6_O_6_S 821.405481, found 821.4027 (Fig. S25). The cargo molecule was first attached to streptavidin in a low molar ratio to avoid occupying the binding sites to a significant amount by mixing 5 μL of 50 μM Atto 565-Biotin with 195 μL of 18.9 μM St to yield 18.4 μM of a non-saturated complex we refer to as St*.

To form a magnetic-field- and pH-responsive DPN (MagDPN), we began to form a [St·BI]_1,2_ DPN around commercially available, streptavidin-functionalized, superparamagnetic beads with a nominal diameter of 1 μm (ThermoFisher Scientific, Product No. 88816, Lot No. YH379063). First, 500 μL of the 10 mg/mL magnetic bead slurry was thrice exchanged into PBS buffer by magnetic pulldown and rinsing the pellet, and the pellet was resuspended in 265 μL PBS, and 35 μL of the magnetic beads was mixed with 35 μL of 18.9 μM St·BI_4_ and incubated at room temperature for 20 minutes. Subsequently, 36 μL of St* was added, followed by another 20-minute incubation. Another 35 μL of 18.9 μM St·BI_4_ was then added, and incubated for 20 minutes. Finally, 36 μL of St* was added, followed by another 20-minute incubation, and then 35 μL of St·BI_4_ was added, followed by a final 20-minute incubation. The result is a MagDPN, which has a total composition of 0.42 μM Atto 565-Biotin, 6.3 μM St, 9.4 μM St·BI_4_, and an estimated 4.9x10^-6^ μM magnetic beads (with 3.2 μM effective streptavidin on the beads) in 212 μL. The relative stoichiometries of this MagDPN were 0.022 Atto 565 per St, 1.989 BI per St, and 3.9x10^6^ St per magnetic bead.

The fluorescence of the Atto565 in the MagDPN sample was monitored using a Cary Eclipse Fluorescence Spectrophotometer (Agilent). Emission intensity at 590 nm was recorded with an excitation wavelength of 565 nm; excitation and emission slit widths were 5 nm; photomultiplier tube voltage was 750 volts; data was averaged for 0.5 s. In this experiment (Fig. 5C), 200 μL of the MagDPN was added to a 350 μL micro-cuvette, and a magnet was placed underneath for 15 seconds. After the magnetic beads were pulled down, the cuvette was placed in the spectrophotometer, and the fluorescence intensity was measured (Fig. S26). The cuvette was removed, 4 μL of 1 M HCl was added and a micropipette was used to mix the solution and resuspend the magnetic beads. A magnet was again placed under the cuvette for 15 seconds, and the cuvette was returned to the spectrophotometer for a second measurement. Finally, 4 μL of 1M NaOH was added, the solution was mixed and the beads were resuspended, the magnetic beads were pulled down with a magnet once more, and a final fluorescence measurement was made. Several control experiments were also performed (Fig. S27). In these samples, the same final compositions of magnetic beads, St*, St, StBI4, and MagDPN were used as specified above. In each case, 200 μL of the specified samples were added to the micro-cuvette, a magnet was placed underneath for 15 seconds to pull down the magnetic beads, and the samples were placed in the fluorometer for a fluorescence intensity measurement.

With these measurements, fluorescence intensity and uncertainty was determined as the mean and standard deviation of three sequential measurements of each sample. All fluorescence intensities were then normalized to the value measured for 0.42 μM Atto 565-Biotin alone. The 80.1% of cargo molecules initially sequestered was calculated as the relative normalized fluorescence intensity for the initial MagDPN *versus* Atto 565-Biotin alone. The 94.0% of sequestered cargo molecules released was calculated as the relative normalized fluorescence intensity for the acidified MagDPN *versus* the initial MagDPN. The 79.7% of re-sequestered cargo molecules was calculated as the relative normalized fluorescence intensity for the neutralized MagDPN *versus* Atto 565-Biotin alone. The 99.5% repeated efficiency was calculated as the relative difference between the re-sequestered cargo molecules *versus* the initially sequestered molecules.

***Supporting Figures***


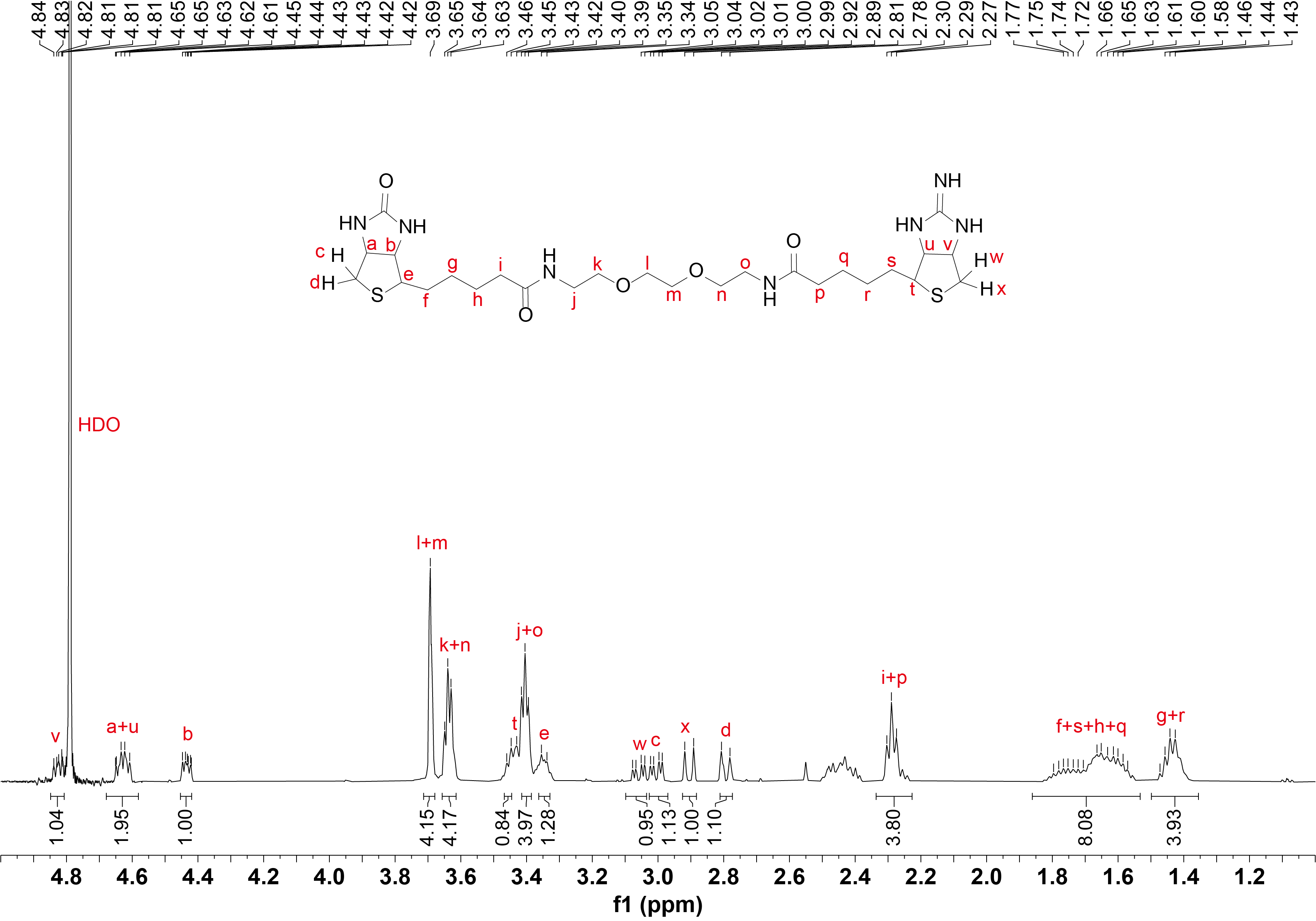


**Figure S1.** ^1^H NMR (D_2_O, 25 °C, 499.9 MHz) spectrum of biotin-PEG_2_-iminobiotin (BI) HAX.

**Figure S2.** Full (A) and zoomed (B) ^13^C NMR (D_2_O, 25 °C, 125.7 MHz) spectrum of biotin-PEG_2_-iminobiotin (BI) HAX.


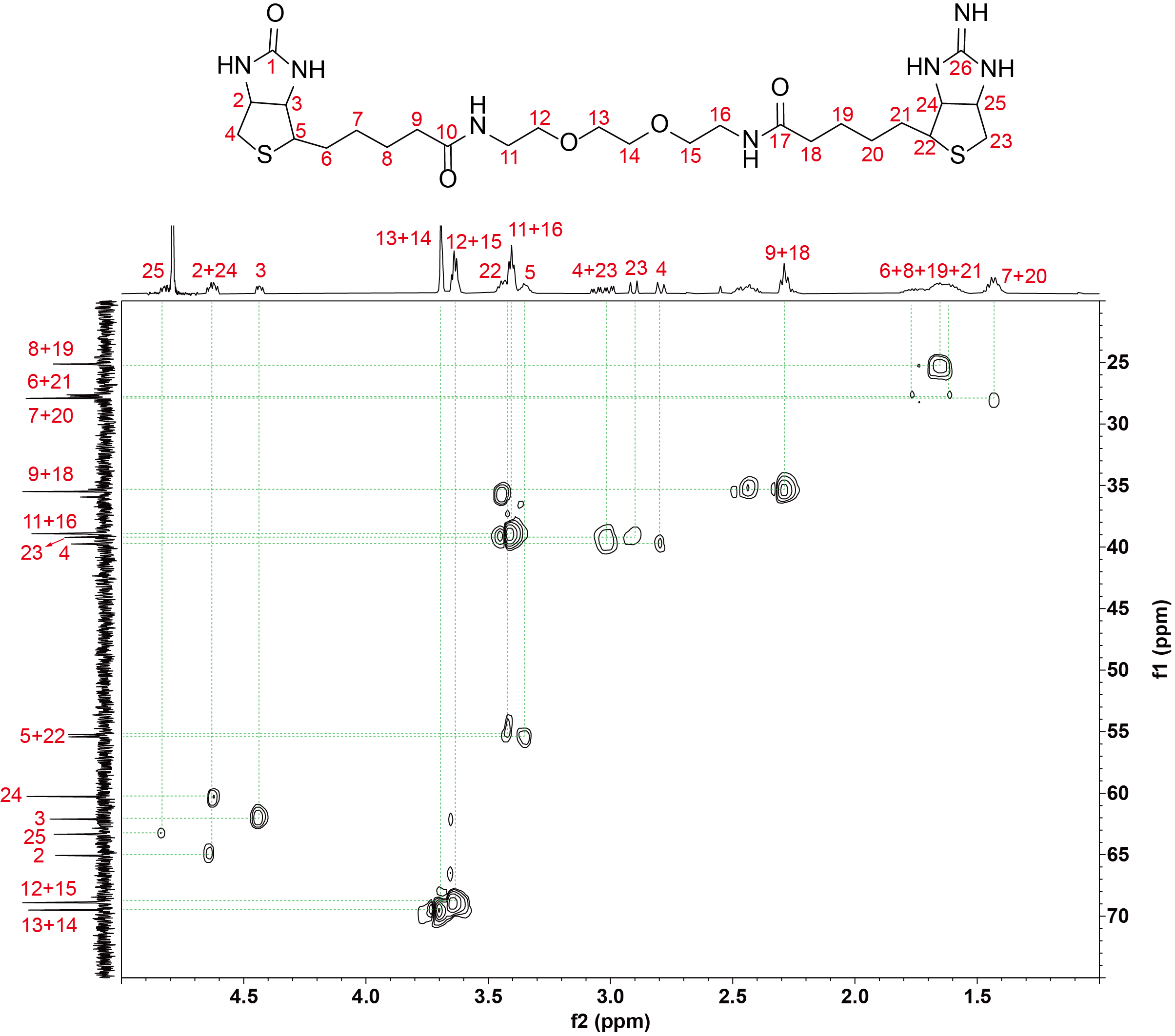


**Figure S3.** Heteronuclear single quantum coherence (^1^H 499.9 MHz, ^13^C 125.7 MHz, D_2_O, 25 °C) spectrum of biotin-PEG_2_-iminobiotin (BI) HAX.


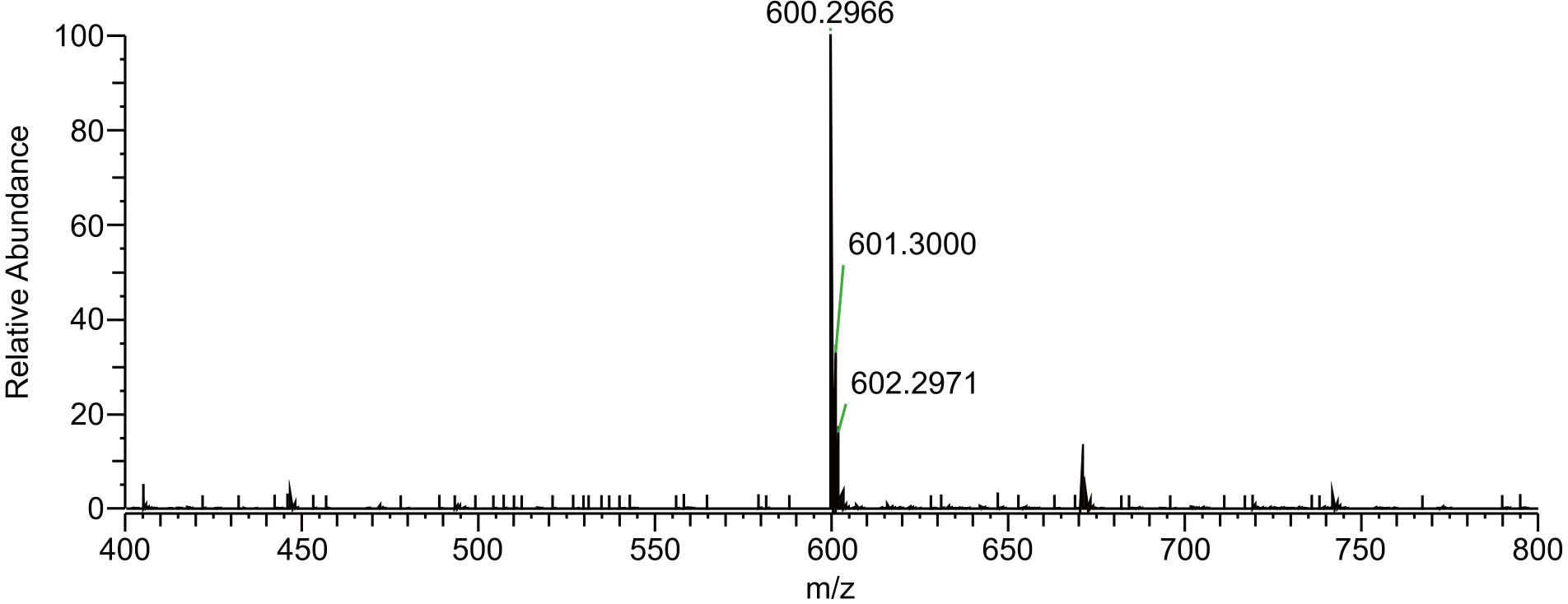


**Figure S4.** HR ESI MS (positive mode) spectrum of biotin-PEG_2_-iminobiotin (BI) HAX. *m/z* [M+H]^+^ calculated for C_26_H_45_N_7_O_5_S_2_ 600.29964, found 600.2966.


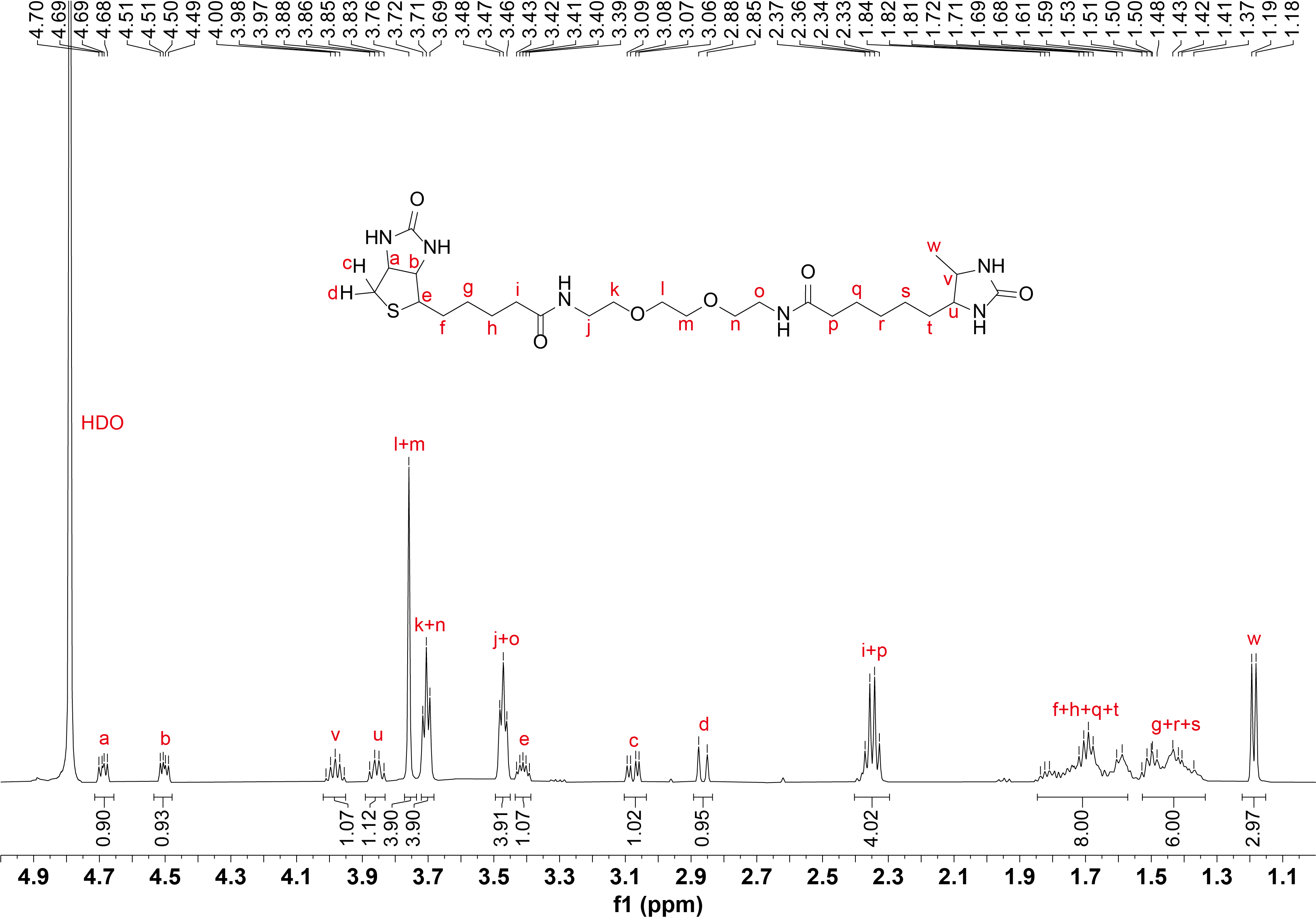


**Figure S5.** ^1^H NMR (D_2_O, 25 °C, 499.9 MHz) spectrum of biotin-PEG_2_-desthiobiotin (BD) HAX.


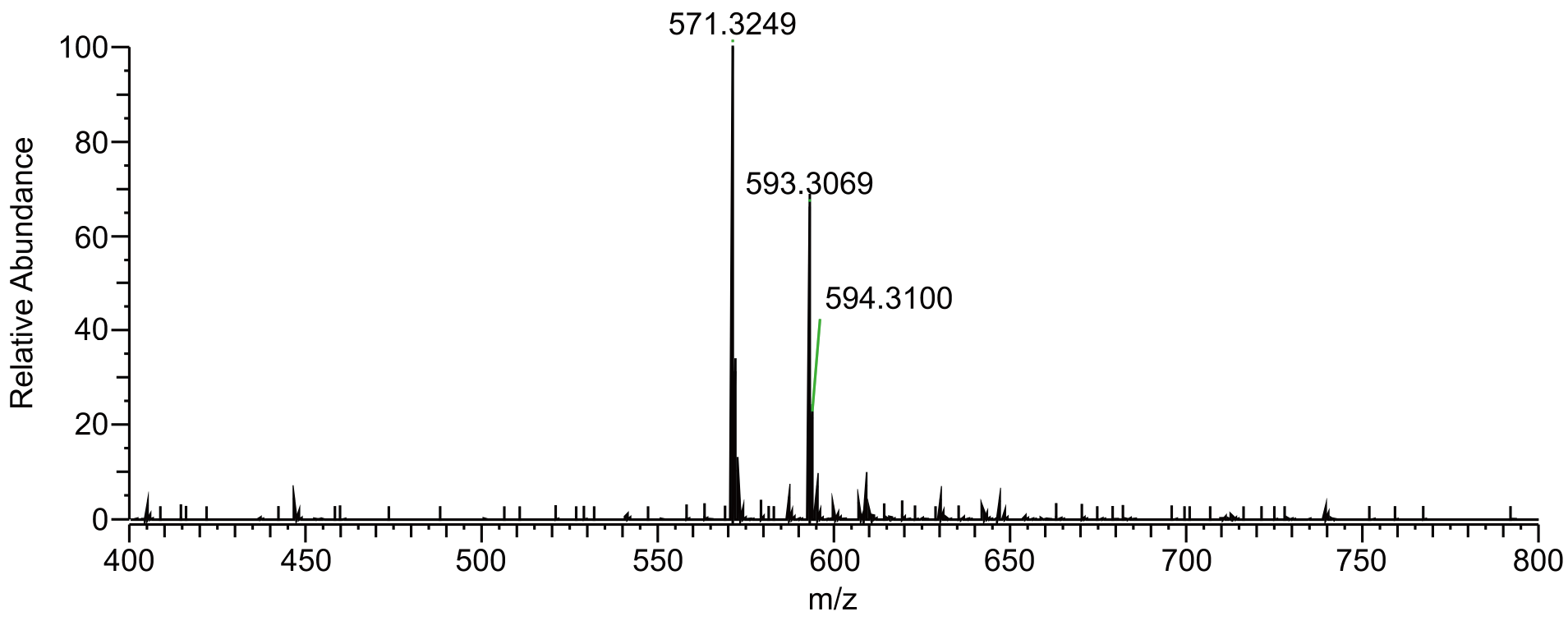


**Figure S6. HR** ESI MS (positive mode) spectrum of biotin-PEG_2_-dethiobiotin (BD) HAX. *m/z* [M+H]^+^ calculated for C_26_H_47_N_6_O_6_S 571.32723, found 571.3249; [M+Na]^+^ calculated for C_26_H_46_N_6_O_6_SNa 593.30917, found 593.3069.


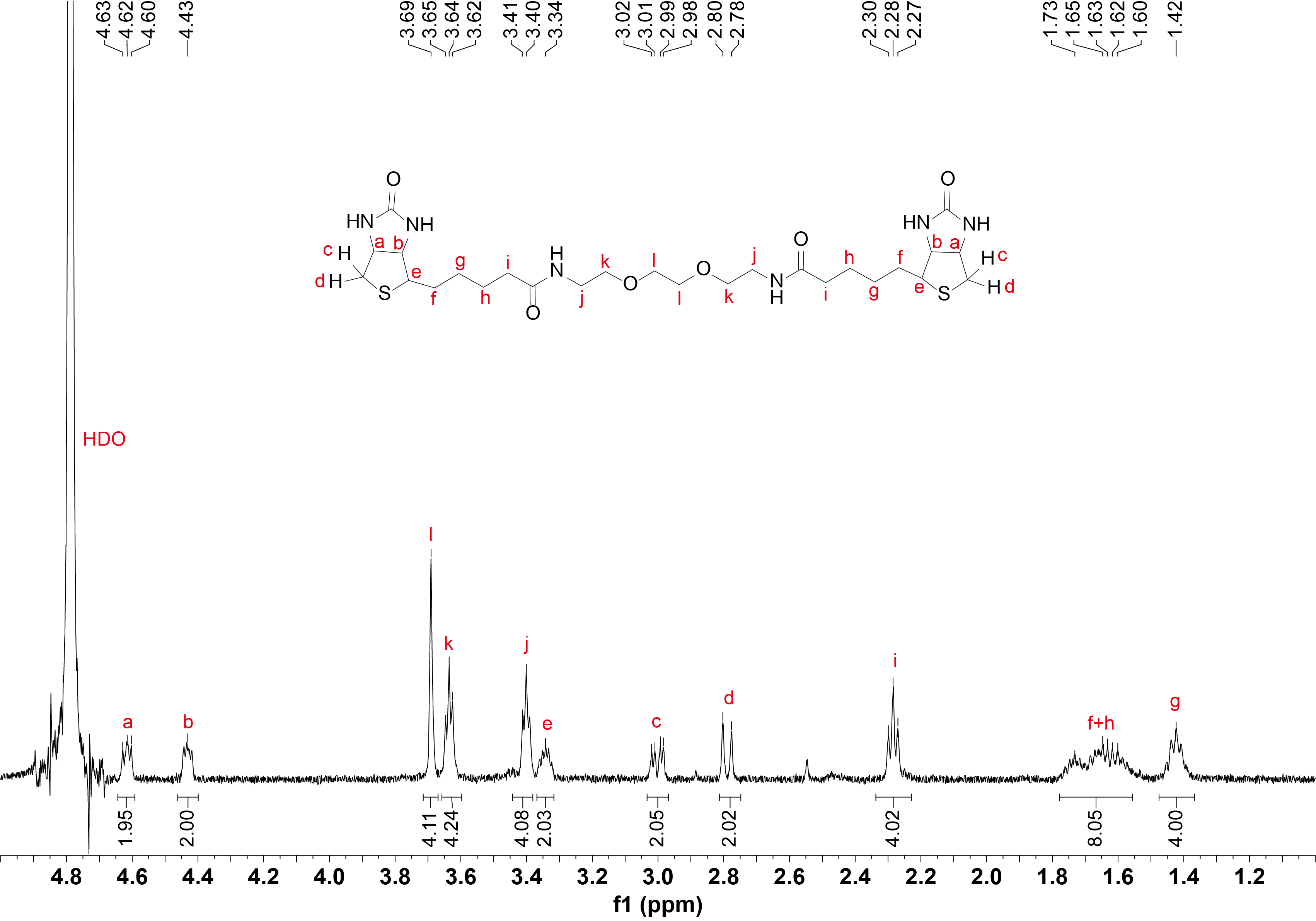


**Figure S7.** ^1^H NMR (D_2_O, 25 °C, 499.9 MHz) spectrum of biotin-PEG_2_-biotin (BB) cross-linker.


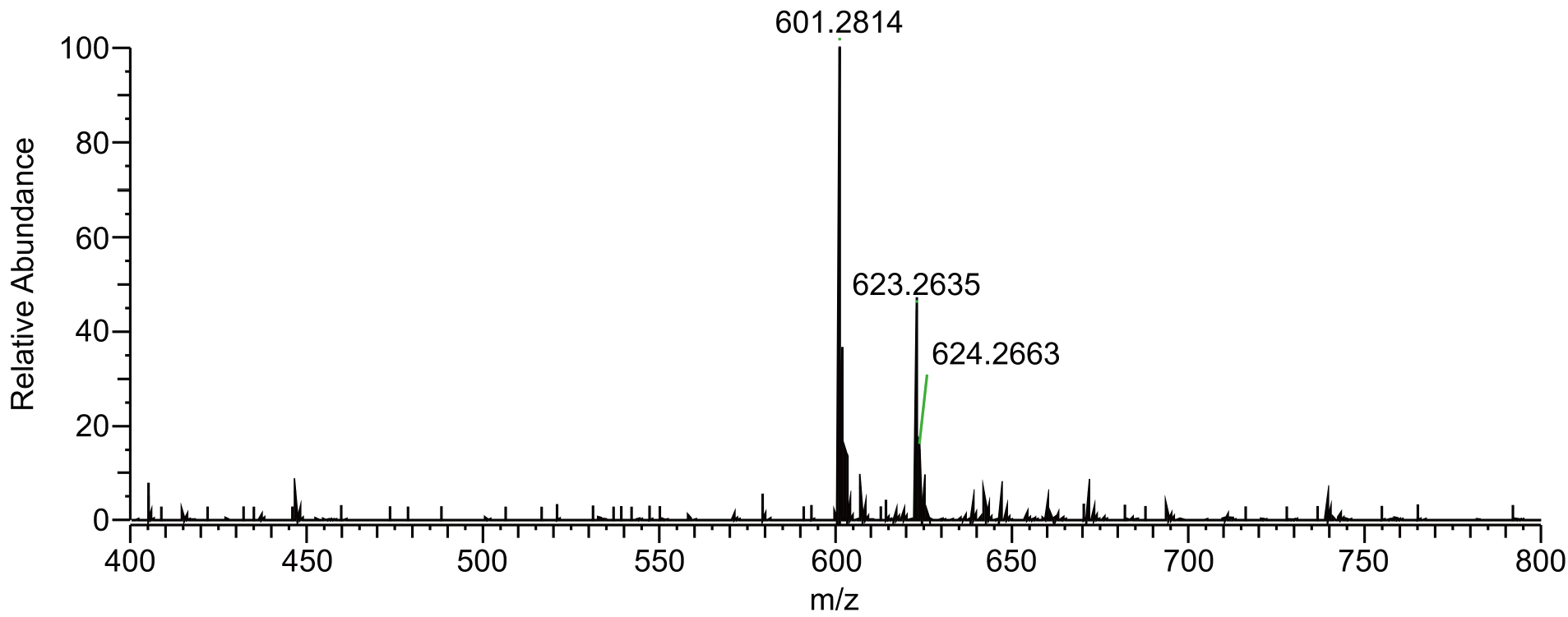


**Figure S8. HR** ESI MS (positive mode) spectrum of biotin-PEG_2_-biotin (BB) cross-linker. *m/z* [M+H]^+^ calculated for C_26_H_44_N_6_O_6_S_2_ 601.28365, found 601.2814; [M+Na]^+^ calculated for C_26_H_44_N_6_O_6_S_2_ 623.26560, found 623.2635.


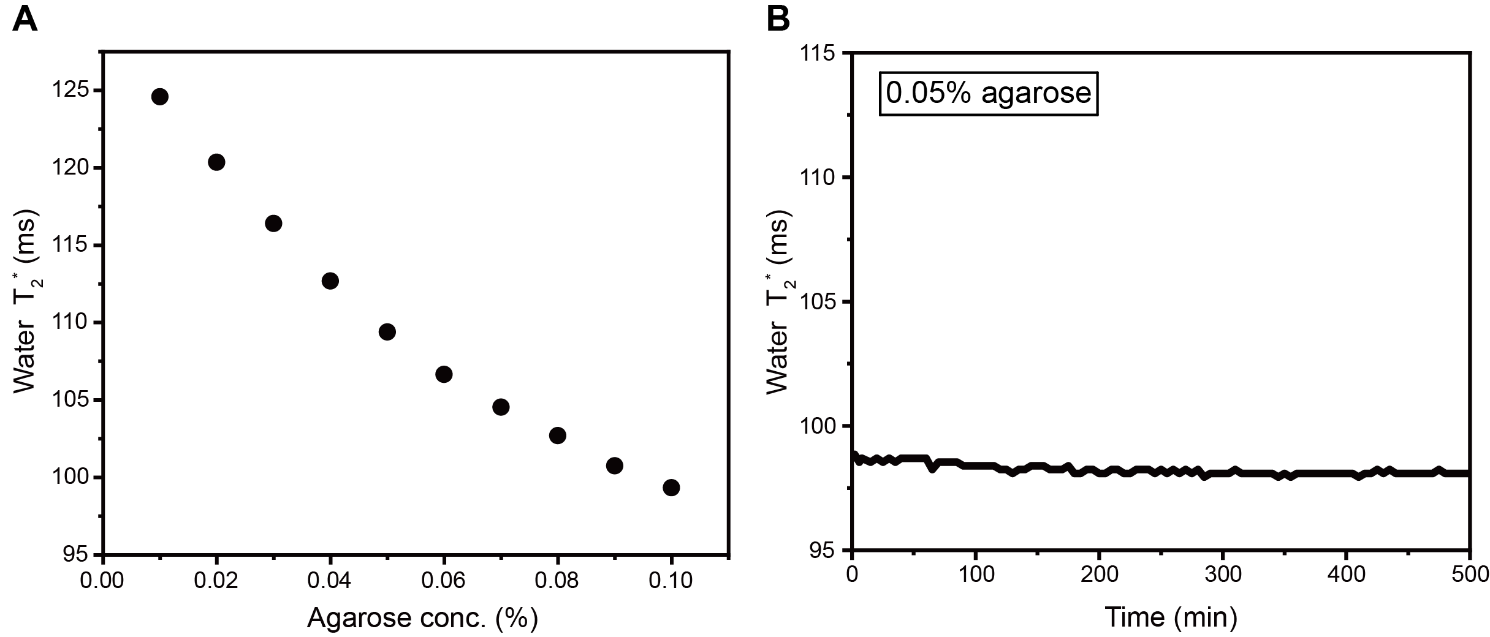


**Figure S9.** Effect of agarose polymer networks on water $T_{2}^{*}$. (A) Agarose concentration-dependence of water $T_{2}^{*}$. Agarose solutions ranging in concentration from 0.01%-0.09% w/v were prepared for a 0.1% w/v stock solution in PBS. (B) Water $T_{2}^{*}$ stability in the presence of a 0.5% w/v agarose polymer network. All agarose solutions were allowed to anneal at room temperature for more than three hours before filling an NMR tube and performing water NMR (see Section 6).


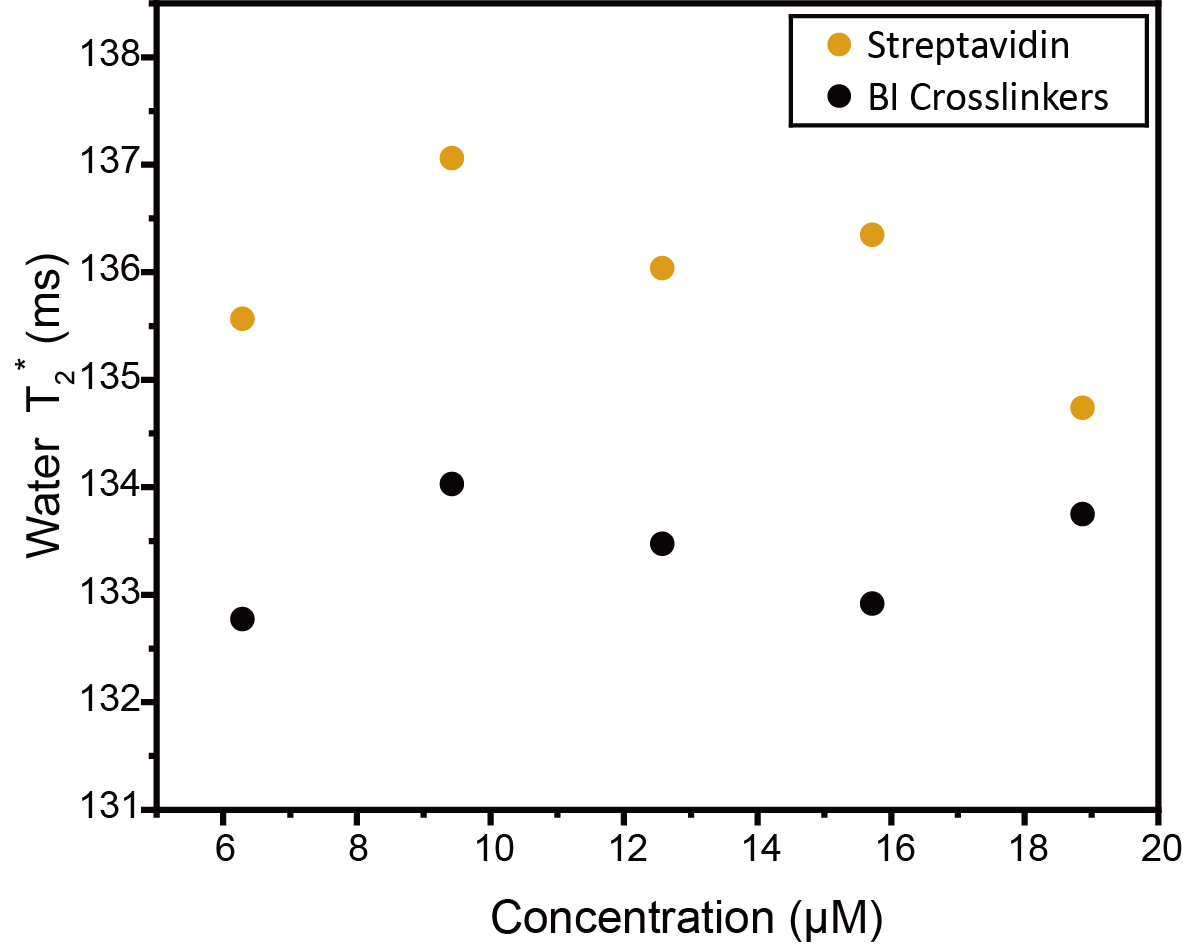


**Figure S10.** Concentration-dependence of water $T_{2}^{*}$ for streptavidin and BI HAX. Solutions were prepared as 300 μL dilutions in PBS buffer pH 7.2 from 1 mg/mL streptavidin (18.87 μM) or 50 μM BI HAX. Water NMR was performed as described in Section 6.


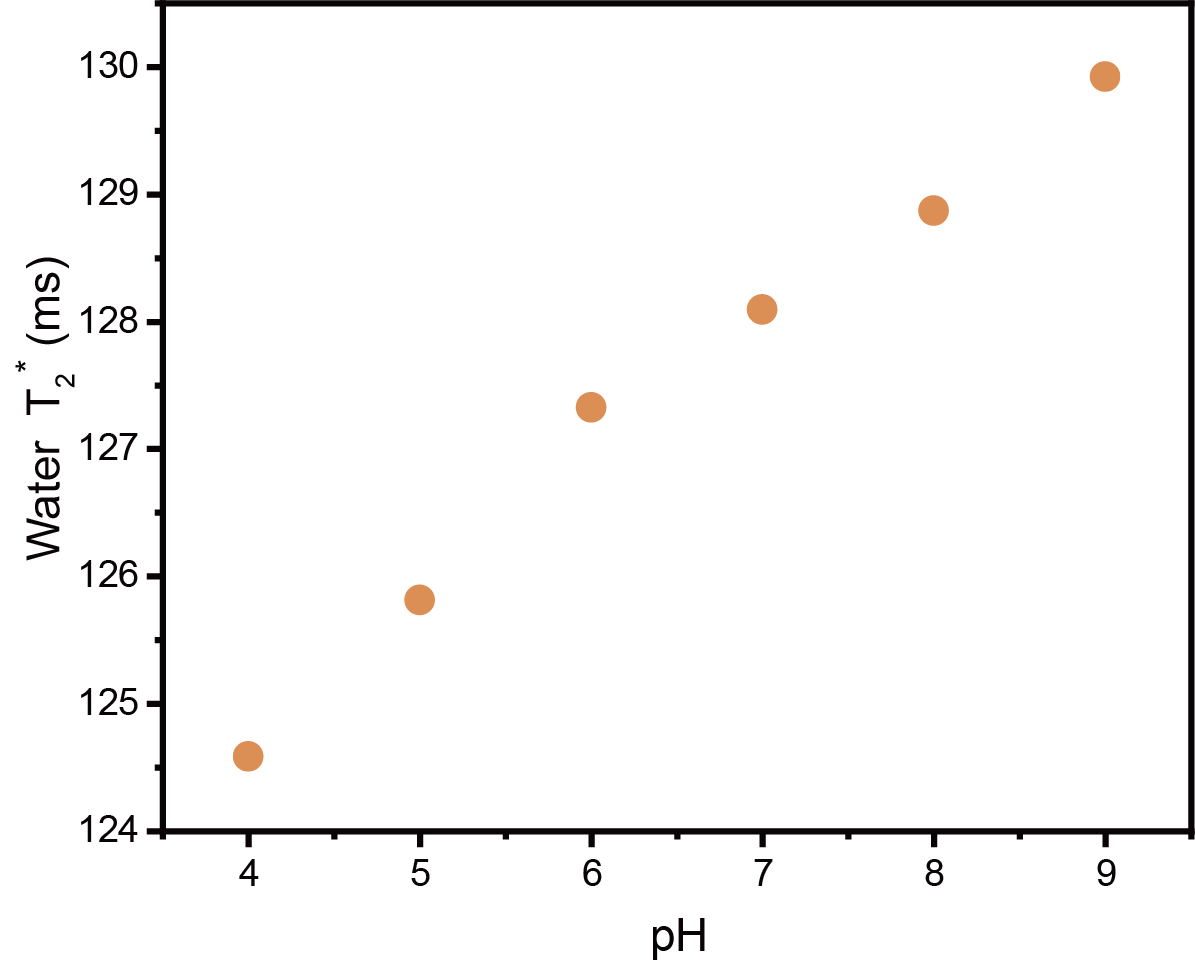


**Figure S11.** pH-dependence of water $T_{2}^{*}$ in the presence of streptavidin. 1 mg/mL streptavidin was solubilized in different buffers depending on pH. Citrate buffer was used for pH 4.0 and 5.0*;* PBS buffer was used for pH 6.0 and pH 7.2, and tris buffer was used for pH 8.0 and 9.0. The pH of each buffer was adjusted by addition of 1 M HCl.

**Figure S12.** Temperature-dependence of water $T_{2}^{*}$ in the presence of a DPN. Temperature was controlled with BSVT control unit. For “heating” (dark colors), temperature was increased every degree between 5 °C to 40 °C, allowed to settle, and a ^1^H NMR (D_2_O, 499.9 MHz) spectrum was acquired as described in Section 6. Once the spectrum at 40 °C was obtained, “cooling” (light colors) occurred in which the temperature was decreased every degree between 40 °C to 5 °C, allowed to settle, and a ^1^H NMR spectrum was acquired. Samples were PBS buffer pH 7.2 (green), 1 mg/mL streptavidin (orange), and a well-equilibrated 1 mg/mL [St·BI]_1,2_ DPN (purple). Both heating and cooling data were simultaneously fit with a first-order polynomial. For PBS buffer, streptavidin, and [St·BI]_1,2_ DPN samples, the slopes for these fits were 2.70, 1.41, and 0.70 ms/°C, respectively, and the R^2^ values were 0.994, 0.999, and 0.986, respectively.


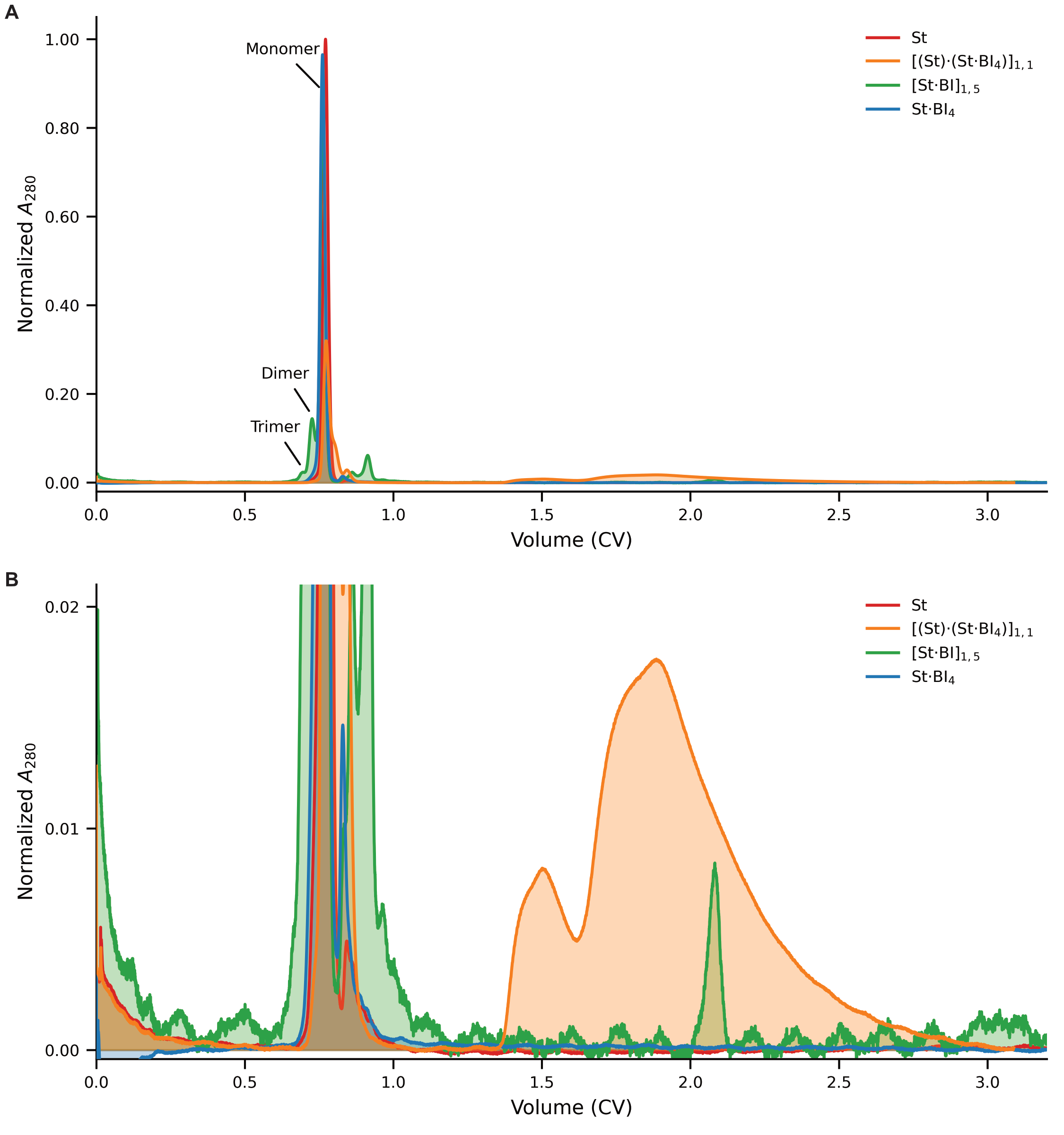


**Figure S13.** Full SEC chromatograms of streptavidin DPNs. A) A_280_ chromatograms for streptavidin (red), streptavidin module (blue), a modular DPN (orange), and a mixture of streptavidin and BI HAX (green). Curves are normalized by total area across chromatogram, and relative to the maximum peak for streptavidin. B) Zoomed in view of chromatograms showing 49.7% of the DPN sample (orange), 5.6% of the mixture (green), and 2.5% of the streptavidin module (blue), and 0.1% of streptavidin (red) exhibits a negative mass shift. Proportion is area between 0.3 to 1 CV *versus* after 1 CV. Spectra are background corrected with a first-order polynomial.


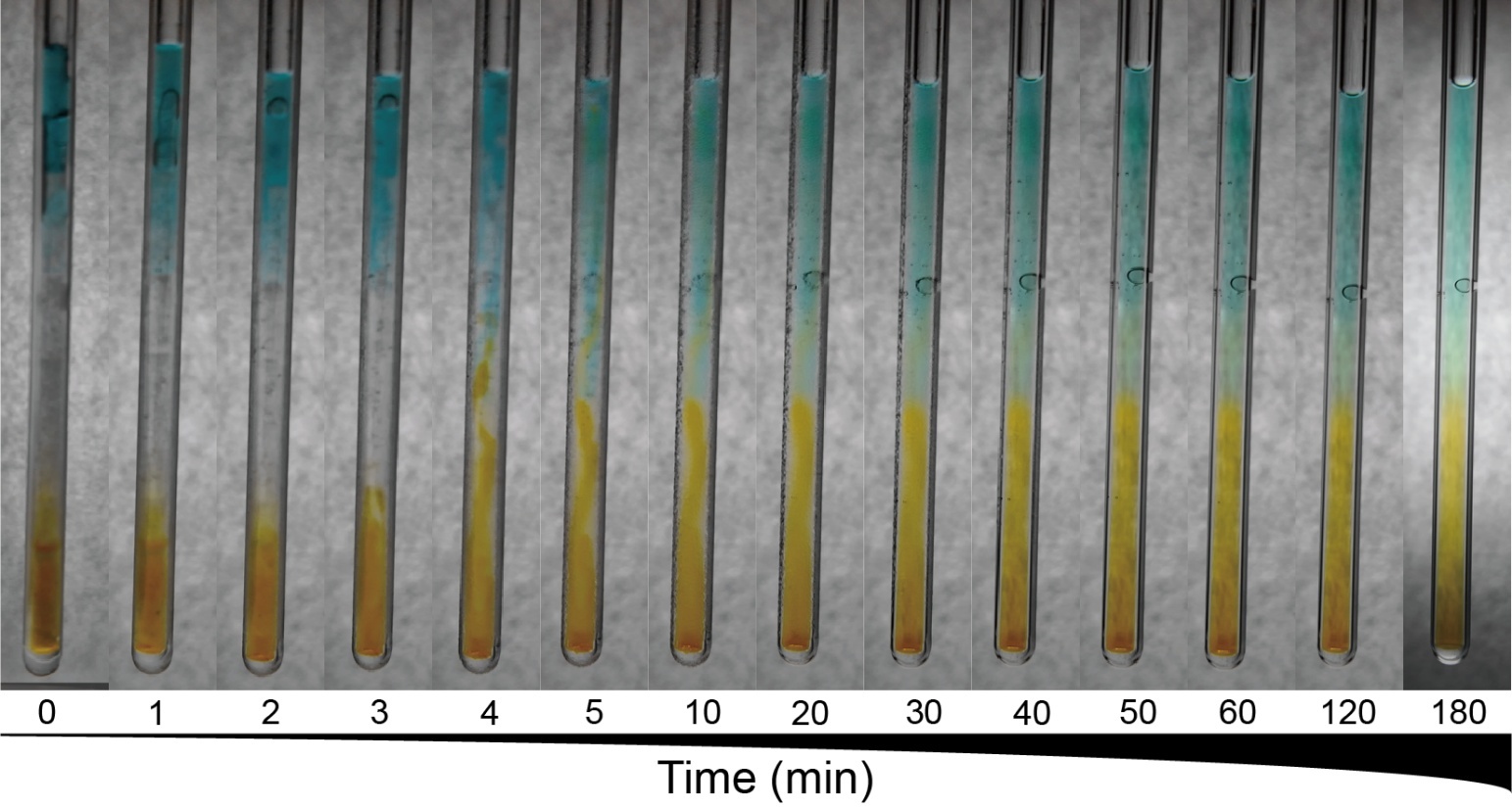
**Figure S14.** Demonstration of cryo-synchronized mixing of food dyes. Reaction was prepared as described in Section 5, using yellow food dye in PBS buffer pH 7.2 for R1, and blue food dye in PBS buffer pH 7.2 for R2. Pictures were taken a cellphone camera at specified times against a background of white paper.


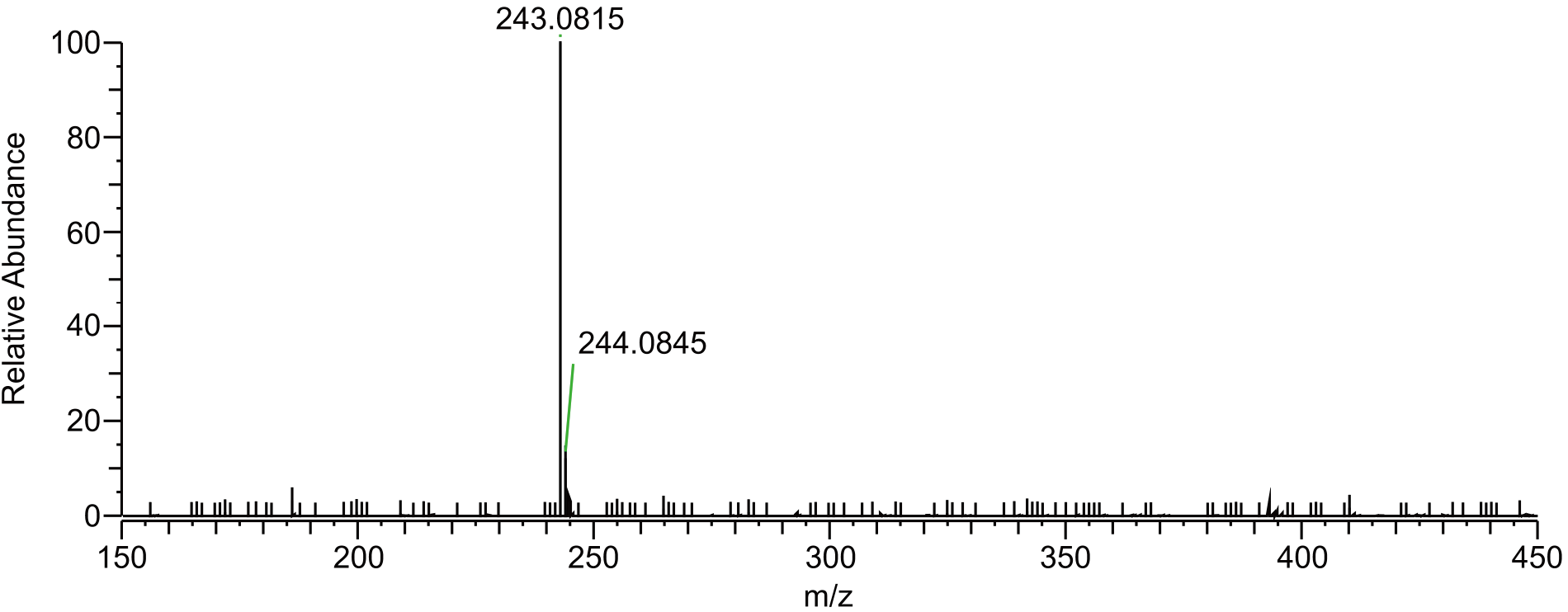


**Figure S15.** HR ESI MS (negative mode) spectrum of biotin**.** *m/z* [M-H]^-^ calculated for C_10_H_15_N_2_O_3_S 243.08089, found 243.0815.

**
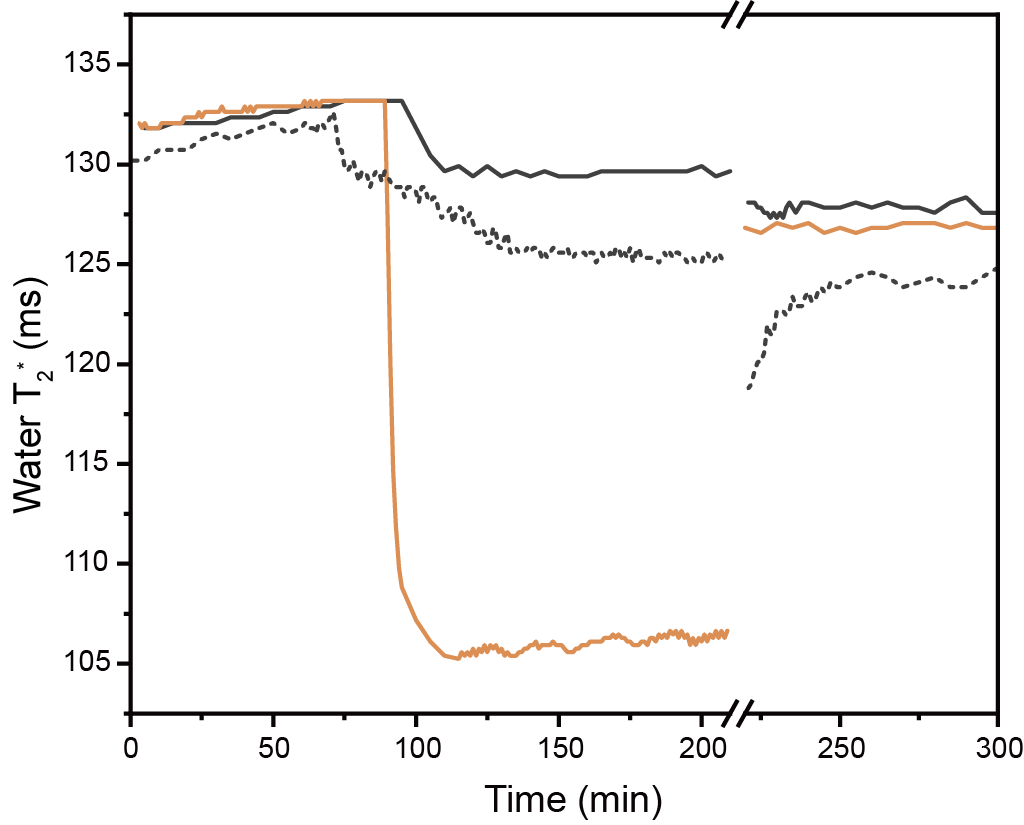
**

**Figure S16.** Replication of cryo-synchronized quenching experiment. Details are similar to those in experiment shown in Fig. 2B: Reaction 1 (1 St, 20 B, 1 St·BI_4_; solid black), Reaction 2 (1 St·BI_4_, 20 B, 1 St; dashed black), Reaction 3 (1 St, 1 St·BI_4_, 20 B; orange). Reaction 2 was performed on a different day and with a different batch of reactants than Reactions 1 and 3.


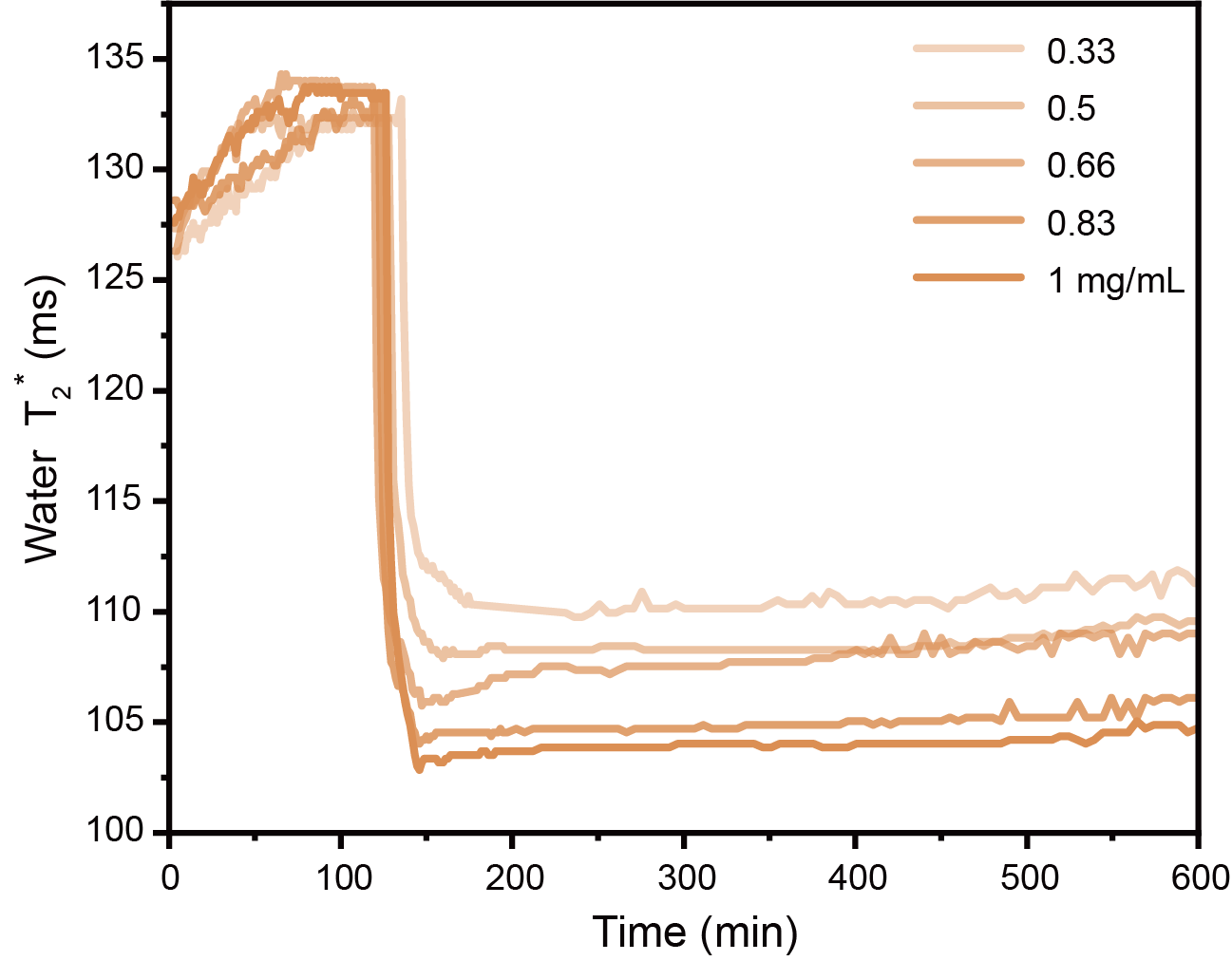


**Figure S17.** Full cryo-synchronized time course of concentration-dependence of [St·BI]_1,2_ DPN formation experiment shown in Fig. 2C for times between 0 to 600 seconds.


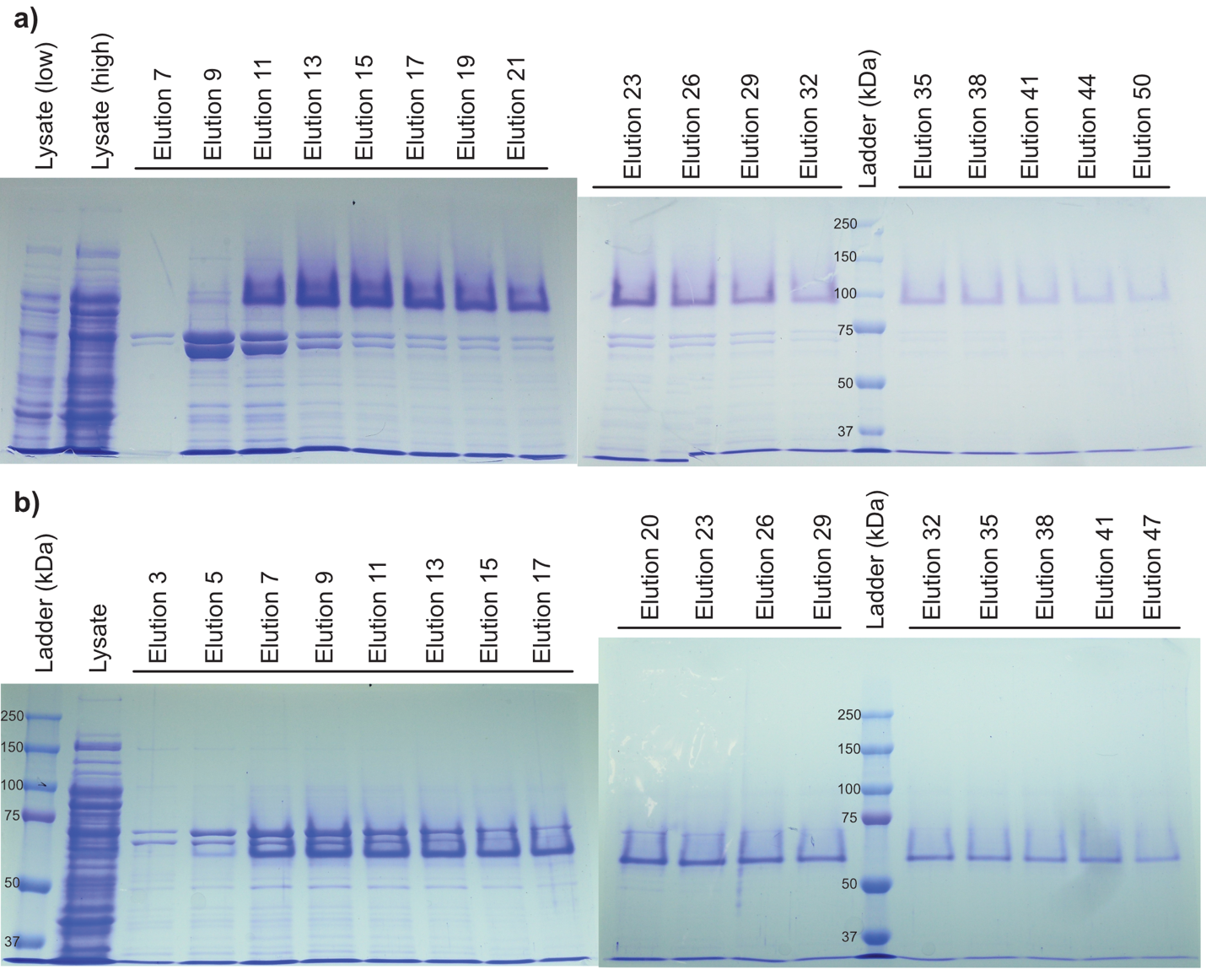
**Figure S18.** Electrophoretic analysis of non-denaturing purification. Annotated images are of 8% SDS-PAGE gels of a typical (a) streptavidin and (b) traptavidin purification. Samples were mixed with SDS-PAGE loading buffer without treatment by boiling. Isocratic elution by imidazole of the hexahistidine-tagged proteins from a Ni-charged nitrilotriacetic acid column was fractionated, and several fractions were loaded in lanes annotated by “Elution #”.


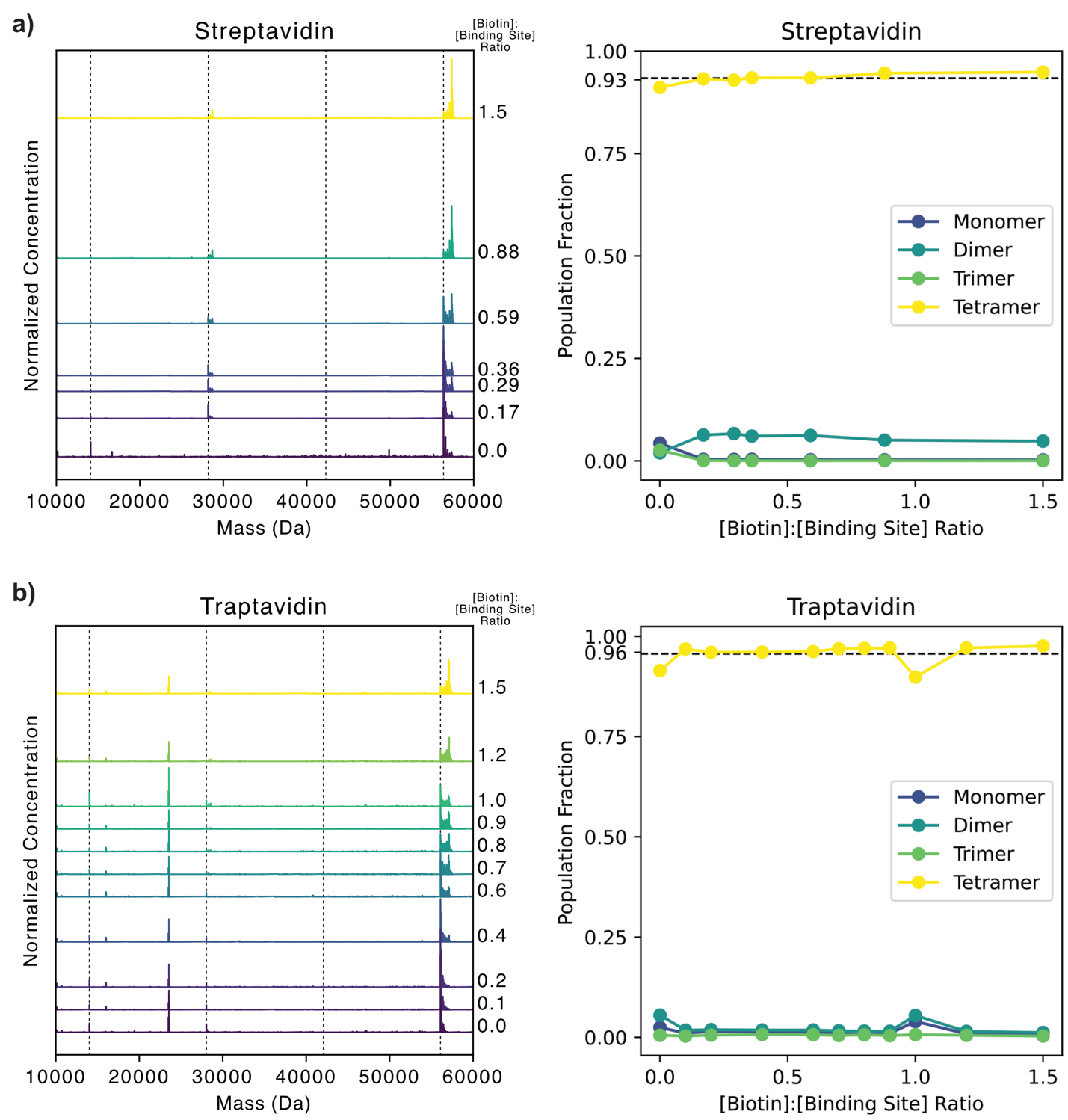
**Figure S19.** Native ESI MS analysis of oligomeric state. (Left) Deconvolved mass spectra of (a) streptavidin and (b) traptavidin with increasing biotin concentrations. Each spectrum is normalized by total area. Dashed lines denote theoretical mass of monomer through tetramer. Theoretical mass from primary sequence with hexahistidine tag and without initial fMet. Streptavidin: predicted 14094.27 Da, observed 14,094 Da; Traptavidin: predicted 14023.14 Da, observed 14,023 Da. (Right) Plot of populations of monomer through tetramer peaks. Values are integrated areas between oligomer mass minus 10 Da through oligomer mass plus mass of maximum number of bound biotins (one through four, 244.31 Da each). Approximately 95% of the avidin-family proteins purified from the soluble fraction were tetrameric.


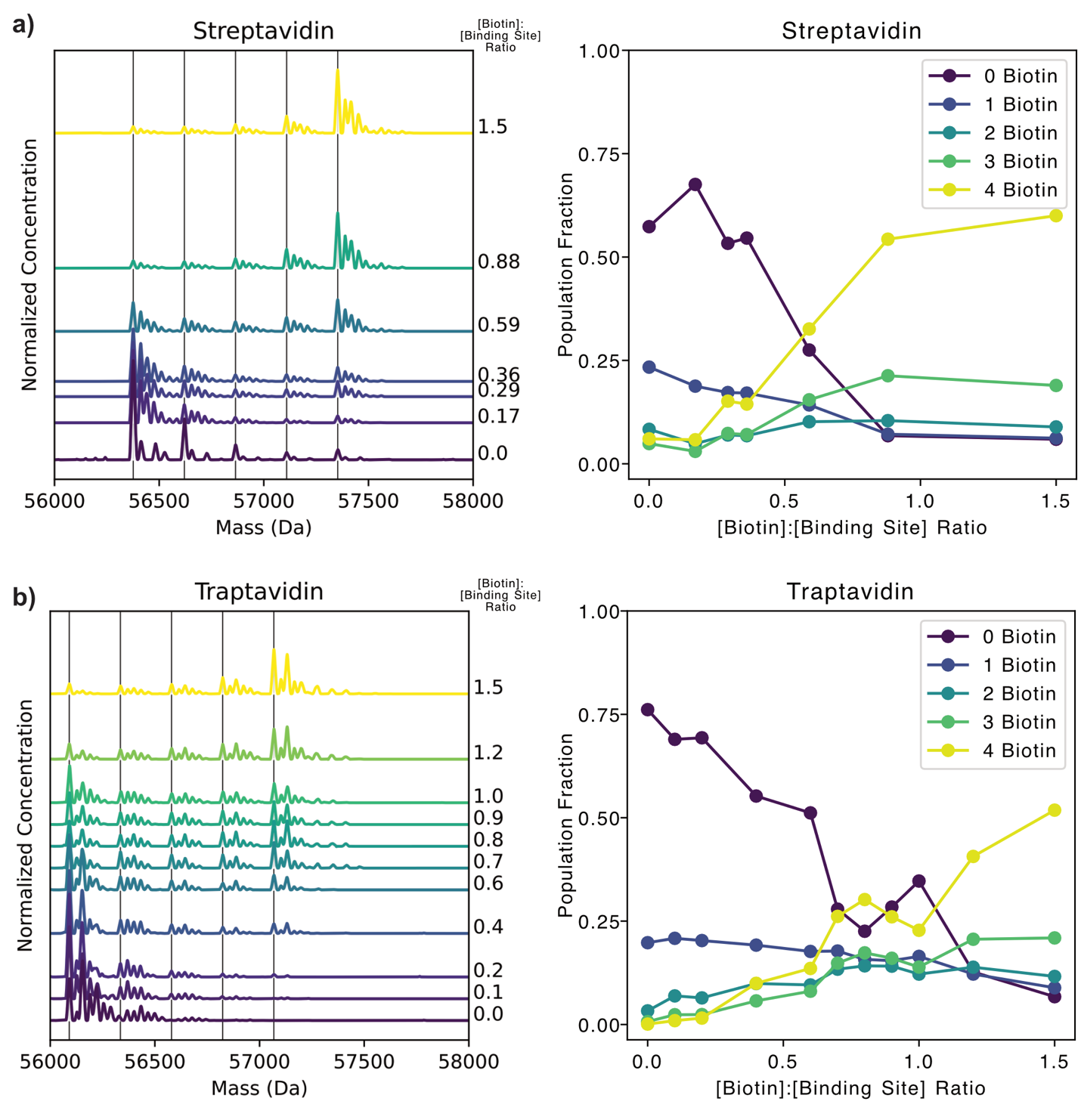


**Figure S20.** Native ESI MS analysis of biotin-binding activity. (Left) Deconvolved mass spectra of (a) streptavidin and (b) traptavidin with increasing biotin concentrations. Each spectrum is normalized by total area. Dashed lines denote mass of apo-tetramer with zero through four bound biotin (244.31 Da) molecules; apo-tetramer mass observed at 56376 Da and 56091 Da for streptavidin and traptavidin, respectively. Binding site concentration determined *via* Bradford assay, and biotin concentration determined by mass. (Right) Plot of populations of biotin-bound proteins. Values are integrated areas between apo-tetramer mass minus 10 Da plus *n* biotins through apo-tetramer mass minus 10 Da plus *n+1* biotins to capture all solvent adducts. Biotin binding exhibits apparent cooperativity and is dependent on the biotin concentration. Only ~6% exhibit no binding, and presence of biotin-bound complexes without addition of exogeneous biotin is due to tight biotin biding during expression and purification.

**
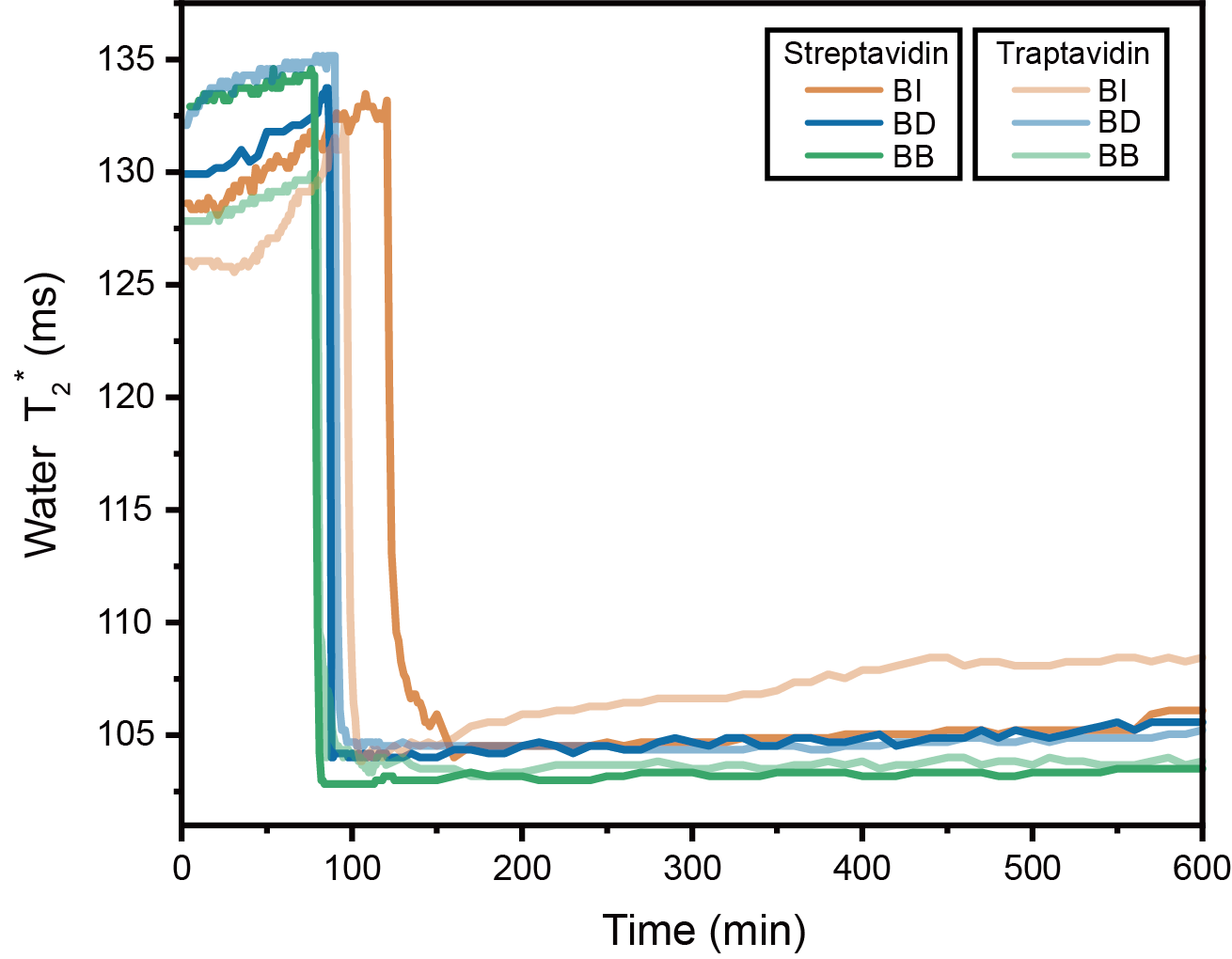
**

**Figure S21.** Full cryo-synchronized time course of DPN formation from different modules as shown in Fig. 3 for times between 0 to 600 seconds.

**
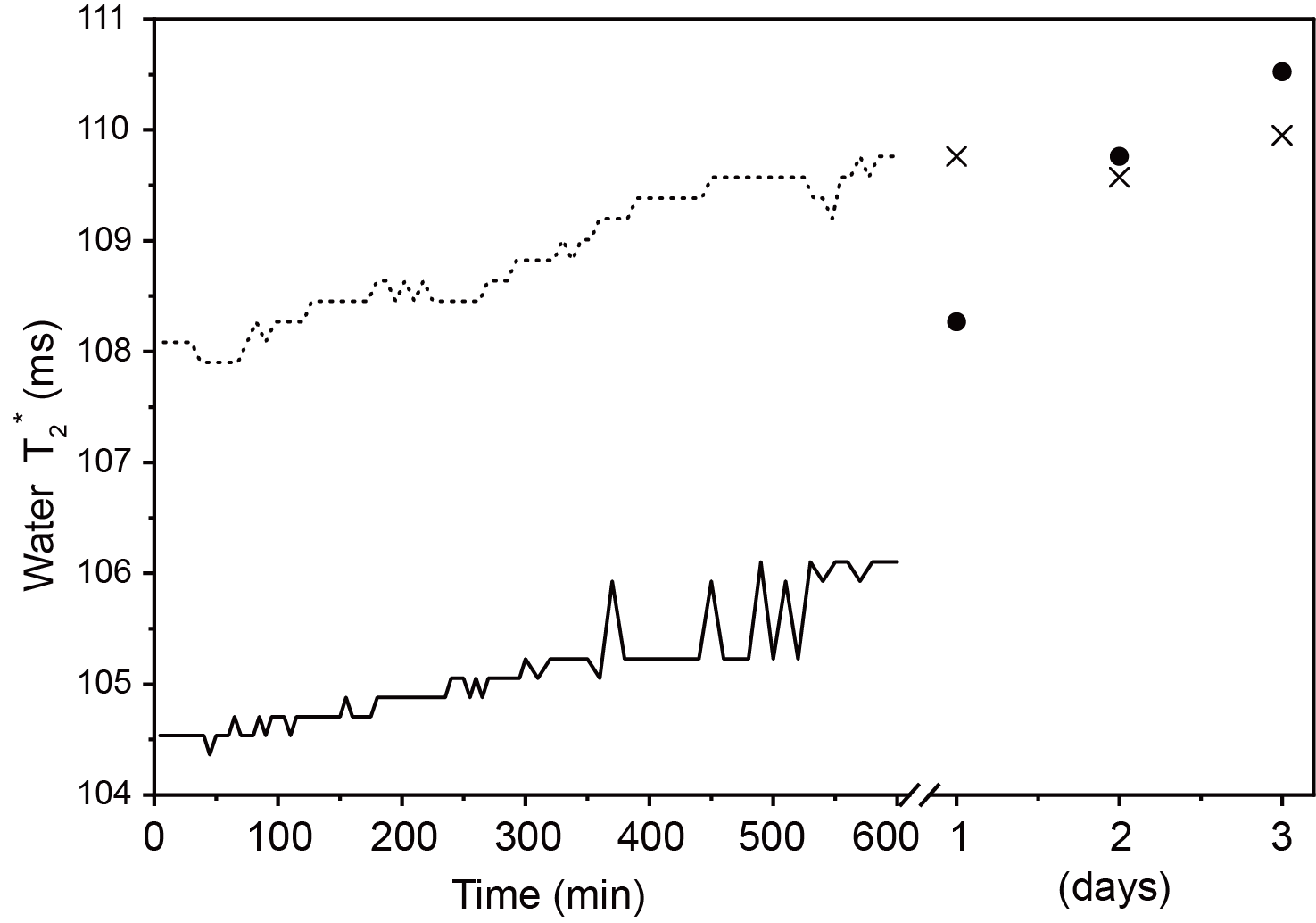
**

**Figure S22.** Replication of modular assembly experiment at high concentrations. Experiment is similar to that in Fig. 4C and described in Section 7E, except was performed here at 18.9 μM of each module for the 1 St + 1 St·BI_4_ (solid) and 1 St·BI_2_ + 1 St·BI_2_ (dashed) reactions only due to low concentrations of pure St·BI_1_.


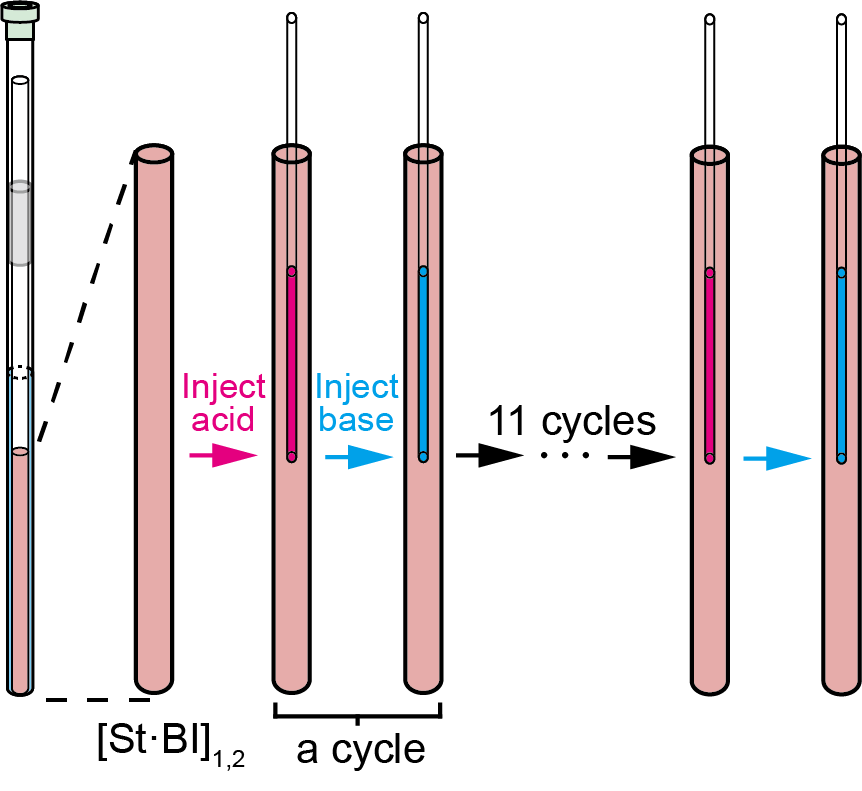


**Figure S23.** Schematic diagram of cycling of pH-sensitive DPN experiment**.** Experimental details are provided in Section 7G.

**
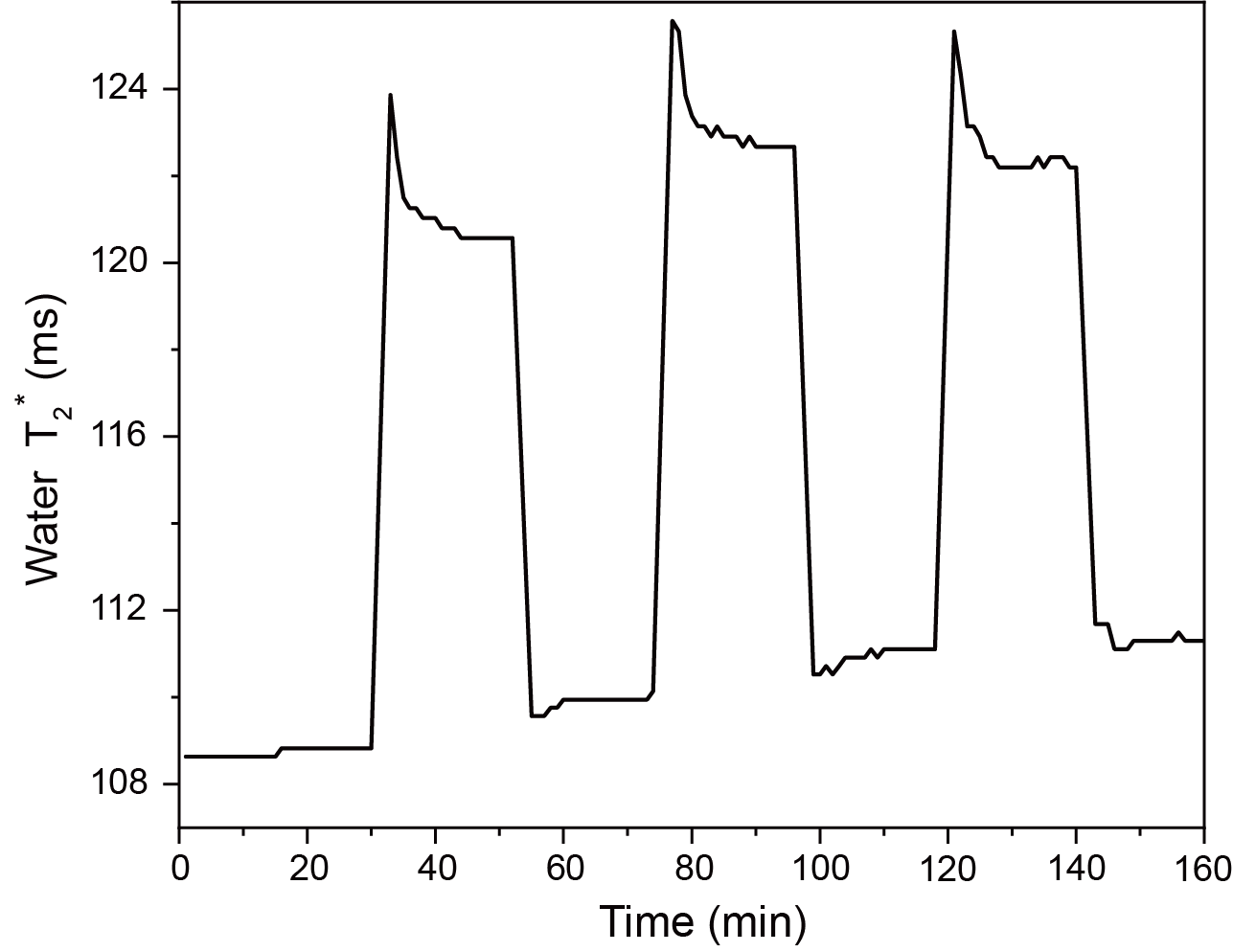
**

**Figure S24.** Three-cycle replication of pH cycling experiment for a [St·BI]_1,2_ DPN. The pH response is the same as in Fig. 5B. Experimental details are provided in Section 7G.


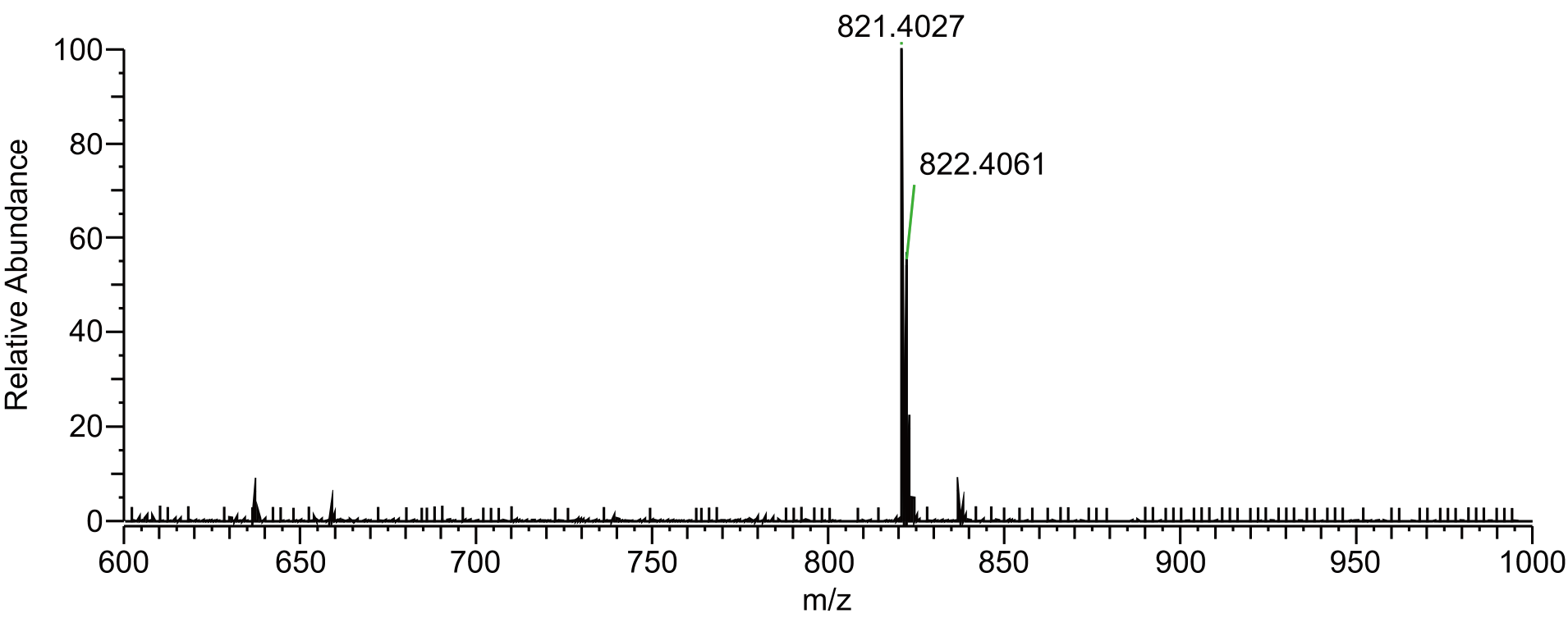


**Figure S25.** ESI MS (positive mode) spectrum of Atto 565-Biotin used in fluorescence experiments as described in Section 7H. HR ESI MS *m/z* [M]^+^ calculated for C_46_H_57_N_6_O_6_S 821.405481, found 821.4027.


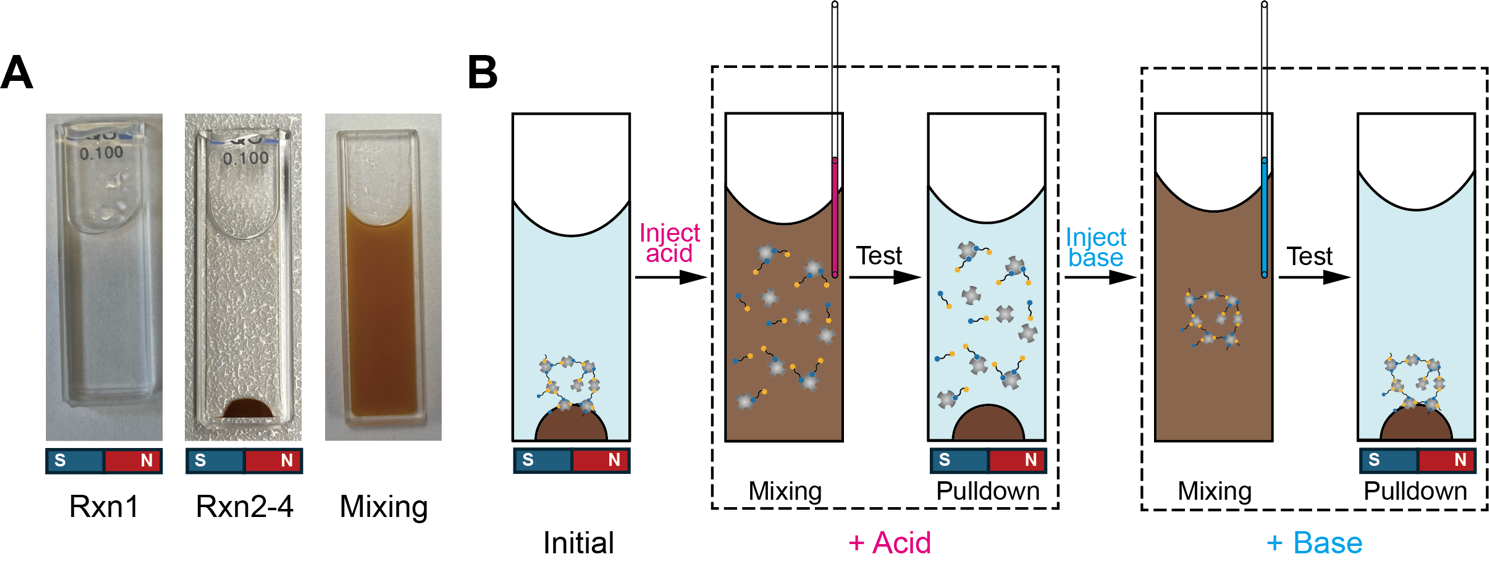


**Figure S26.** Schematic diagram of controlled molecular delivery experiment performed using pH-sensitive DPN on a fluorescence spectrophotometer. (A) Example photographs showing the cuvettes with fluorophores but no magnetic beads (Rxn1), fluorophores with magnetic beads pulled down (Rxn2-4), and magnetic beads before any magnetic pull-down (Mixing). (B) Schematic of ordered steps of pH-response experiment in Fig. 5C. Experimental details are provided in Section 7H. The BI-based DPN associated with a pellet of magnetic beads is disassembled upon addition of acid, and protein modules can no longer be pulled down with a magnet. The DPN reassembles upon addition of base and can once again be pulled down with a magnet.


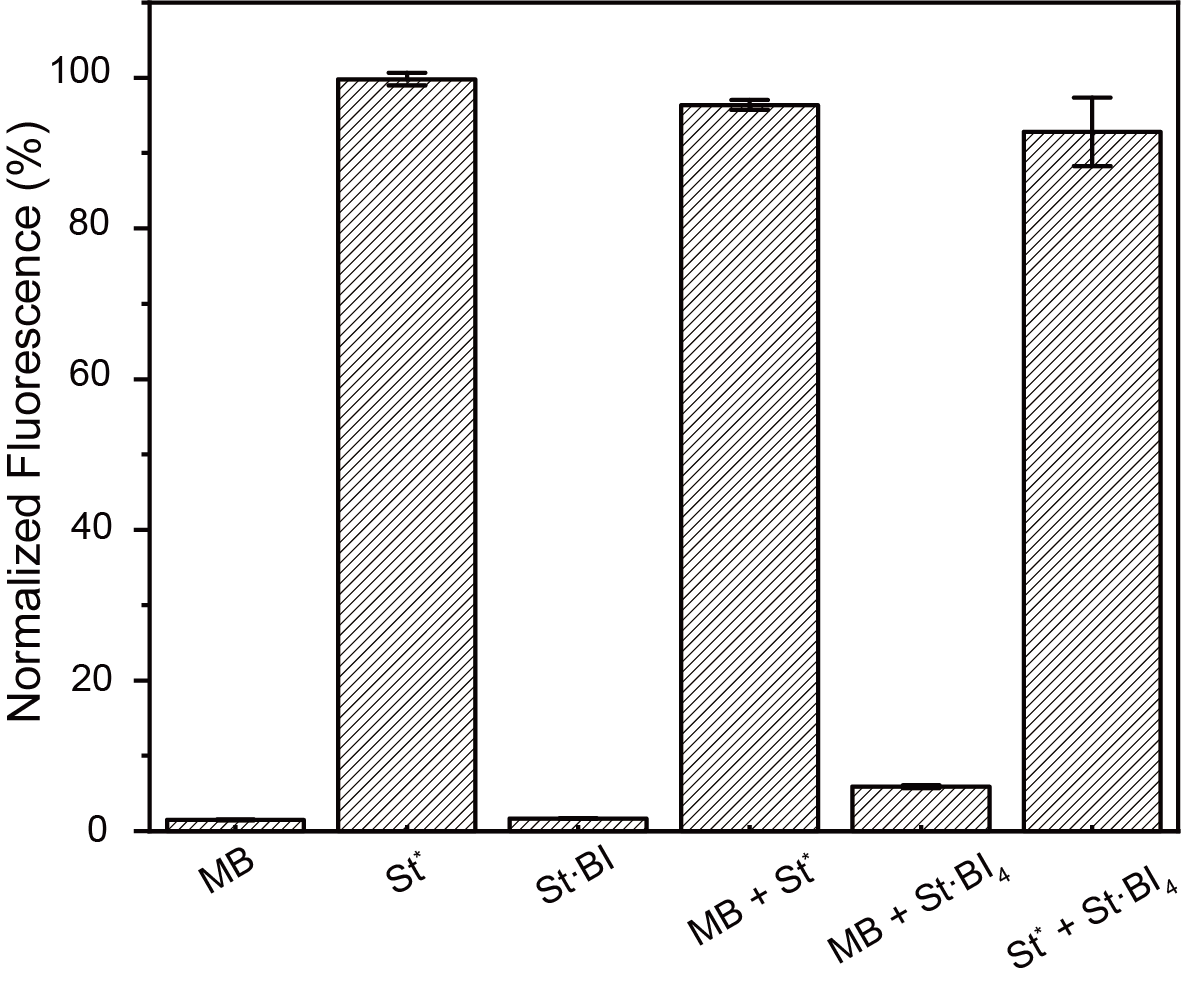


**Figure S27.** Controls for controlled molecular delivery experiment performed using pH-sensitive DPN on a fluorescence spectrophotometer. Experimental details are provided in Section 7H. Abbreviation: Magnetic Beads, MB; St*, sub-stoichiometric amount of Atto 565-biotin bound to streptavidin.

***Supporting References***

[1] M. Howarth, D. J.-F. Chinnapen, K. Gerrow, P. C. Dorrestein, M. R. Grandy, N. L. Kelleher, A. El-Husseini, A. Y. Ting, “A monovalent streptavidin with a single femtomolar biotin binding site” *Nature Methods* **2006**, *3*, 267–273.

[2] L. Konermann, “Addressing a Common Misconception: Ammonium Acetate as Neutral pH ‘Buffer’ for Native Electrospray Mass Spectrometry” *Journal of the American Society for Mass Spectrometry* **2017**, *28*, 1827–1835.

[3] D. P. Donnelly, C. M. Rawlins, C. J. DeHart, L. Fornelli, L. F. Schachner, Z. Lin, J. L. Lippens, K. C. Aluri, R. Sarin, B. Chen, C. Lantz, W. Jung, K. R. Johnson, A. Koller, J. J. Wolff, I. D. G. Campuzano, J. R. Auclair, A. R. Ivanov, J. P. Whitelegge, L. Paša-Tolić, J. Chamot-Rooke, P. O. Danis, L. M. Smith, Y. O. Tsybin, J. A. Loo, Y. Ge, N. L. Kelleher, J. N. Agar, “Best practices and benchmarks for intact protein analysis for top-down mass spectrometry” *Nature Methods* **2019**, *16*, 587–594.

[4] M. Källsten, D. Visanu, M. Pijnappel, F. Lehmann, J. Bergquist, S. B. Lind, L. Kovac, “Potential Use of Supercharging Agents for Improved Mass Spectrometric Analysis of Monoclonal Antibodies and Antibody–Drug Conjugates” *Journal of the American Society for Mass Spectrometry* **2022**, *33*, 1161–1167.

[5] M. T. Marty, A. J. Baldwin, E. G. Marklund, G. K. A. Hochberg, J. L. P. Benesch, C. V. Robinson, “Bayesian Deconvolution of Mass and Ion Mobility Spectra: From Binary Interactions to Polydisperse Ensembles” *Analytical Chemistry* **2015**, *87*, 4370–4376.
